# MRI‐Compatible and Conformal Electrocorticography Grids for Translational Research

**DOI:** 10.1002/advs.202003761

**Published:** 2021-03-08

**Authors:** Florian Fallegger, Giuseppe Schiavone, Elvira Pirondini, Fabien B. Wagner, Nicolas Vachicouras, Ludovic Serex, Gregory Zegarek, Adrien May, Paul Constanthin, Marie Palma, Mehrdad Khoshnevis, Dirk Van Roost, Blaise Yvert, Grégoire Courtine, Karl Schaller, Jocelyne Bloch, Stéphanie P. Lacour

**Affiliations:** ^1^ Bertarelli Foundation Chair in Neuroprosthetic Technology Laboratory for Soft Bioelectronic Interfaces Institute of Microengineering Institute of Bioengineering Center for Neuroprosthetics Ecole Polytechnique Fédérale de Lausanne (EPFL) Geneva 1202 Switzerland; ^2^ Department of Neurosurgery University Hospital of Lausanne (CHUV) and University of Lausanne (UNIL) Lausanne 1010 Switzerland; ^3^ Defitech Center for Interventional Neurotherapies (NeuroRestore) Department of Neurosurgery University Hospital of Lausanne (CHUV) University of Lausanne (UNIL) Lausanne 1015 Switzerland; ^4^ UPCourtine Center for Neuroprosthetics and Brain Mind Institute School of Life Sciences Ecole Polytechnique Fédérale de Lausanne (EPFL) Geneva 1202 Switzerland; ^5^ Department of Neurosurgery Hôpital Universitaire de Genève (HUG) Geneva 1205 Switzerland; ^6^ BrainTech Laboratory Inserm Univ Grenoble Alpes Grenoble 38400 France; ^7^ Department of Neurosurgery Ghent University Ghent 9000 Belgium; ^8^ Present address: Institut des Maladies Neurodégénératives – CNRS UMR 5293 Université de Bordeaux Centre Broca Nouvelle‐Aquitaine 146 rue Léo Saignat – CS 61292 – Case 28, Bordeaux cedex Bordeaux 33076 France

**Keywords:** electrocorticography, MRI compatibility, neural implants, soft electrodes, translational research

## Abstract

Intraoperative electrocorticography (ECoG) captures neural information from the surface of the cerebral cortex during surgeries such as resections for intractable epilepsy and tumors. Current clinical ECoG grids come in evenly spaced, millimeter‐sized electrodes embedded in silicone rubber. Their mechanical rigidity and fixed electrode spatial resolution are common shortcomings reported by the surgical teams. Here, advances in soft neurotechnology are leveraged to manufacture conformable subdural, thin‐film ECoG grids, and evaluate their suitability for translational research. Soft grids with 0.2 to 10 mm electrode pitch and diameter are embedded in 150 µm silicone membranes. The soft grids are compatible with surgical handling and can be folded to safely interface hidden cerebral surface such as the Sylvian fold in human cadaveric models. It is found that the thin‐film conductor grids do not generate diagnostic‐impeding imaging artefacts (<1 mm) nor adverse local heating within a standard 3T clinical magnetic resonance imaging scanner. Next, the ability of the soft grids to record subdural neural activity in minipigs acutely and two weeks postimplantation is validated. Taken together, these results suggest a promising future alternative to current stiff electrodes and may enable the future adoption of soft ECoG grids in translational research and ultimately in clinical settings.

## Graphical abstract

Current clinical electrocorticography (ECoGs) grids are made from stiff materials and only offer sparse brain sampling during epilepsy or tumor surgeries. Here conformable ECoG grids made from silicone membranes permit easy implantation in hidden anatomical locations in cadaveric specimen and imaging compatibility with 3T magnetic resonance imaging. In vivo in a minipig model the conformable ECoG grids show high‐resolution brain recordings.

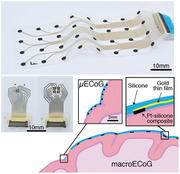

## 1 Introduction

Functional neurosurgeons today routinely employ implantable electrodes to help assessing and treating neurological and traumatic disorders.^[^ ^1^, ^2^, ^3^, ^4^, ^5^, ^6^, ^7^ ^]^ Epidural or subdural cortical grids, here referred as electrocorticography grids (ECoGs), are a class of such electrodes that are mainly used to collect intra‐ or extraoperatively (up to 30 days, the patient staying at the hospital) functional maps of cortical activity with high spatiotemporal resolution, ahead of resection of brain tumors or epileptic foci. More recently, ECoGs are also being evaluated in neuroprosthetic applications to restore motor functions,^[^ ^8^ ^]^ modulate pain,^[^ ^9^ ^]^ alleviate epilepsy in a closed loop system^[^ ^10^ ^]^ or encode then decode speech representation,^[^ ^11^ ^]^ among others.

Current clinical ECoGs host bulk metal stainless steel or Pt–Ir (platinum–iridium) disk electrodes as recording or stimulation sites, soldered individually to discrete wires further embedded in a ≈1 mm thick “rigid” silicone envelope.^[^ ^12^ ^]^ Recent industrialization and automation of this materials system allow for refined electrode layouts using 25 µm thick Pt–Ir sheets instead.^[^ ^13^ ^]^ Electrodes are usually 2.3 mm in diameter with a 10 mm pitch and are arranged in 2 to 8 strips or 8 to 32 grids; denser electrode layouts are also becoming available for clinical research (an overview of current devices with the corresponding parameters is available in Table S1, Supporting Information). Clinical ECoGs may be bent or partly cut to cover the cortical region of interest, including areas that are hidden from the surface such as the occipital lobe or the interhemispheric fissure. However, because their effective bending stiffness is high and dominated by the rigid metal components and the thick silicone shell, poor electrode‐cortex contact is observed, undesirable electrode movement may ensue,^[^ ^14^ ^]^ and adverse effects are observed,^[^ ^15^ ^]^ thereby restricting ECoG use.

Most neurosurgical procedures require pre‐ and postsurgery imaging, magnetic resonance imaging (MRI) being a technique of choice for brain imaging. Unfortunately, implanted ECoGs pose challenges to MRI imaging, given the artifacts that surround and blur the electrode contacts and the adverse risks of tissue heating surrounding the metallic electrodes.^[^ ^16^ ^]^ This precludes additional diagnostics in the surgical theatre and post‐operative imaging, or subsequent functional imaging.^[^ ^16^, ^17^ ^]^

From an opposite perspective, many surgical protocols are shaped around the technological limits of the electrodes that are currently available and their safety or usability restrictions. We therefore explored opportunities based on advances in materials and medical engineering, and engaged with an interdisciplinary team of engineers, neuroscientists and functional neurosurgeons to propose customizable and conformable grids suitable for translational research and novel surgical approaches.

The past decade has witnessed significant development in thin‐film and plastic‐based electrode arrays.^[^ ^18^ ^]^ Micro‐ECoGs on thin (1–100 µm) polymer carriers can be prepared with a variety of inorganic and organic thin films to host up to 240 channels with densities of up to 64 microelectrodes per mm^2^.^[^ ^19^, ^20^, ^21^, ^22^, ^23^, ^24^ ^]^ These micro‐ECoGs are usually tested in preclinical models, at times in clinical investigations; recent results demonstrated the possibility of measuring fine neural activity unraveling neural correlates at the cellular level directly from the surface of the cortex without penetrating it.^[^ ^25^, ^26^, ^27^, ^28^ ^]^ Advanced designs based on multiplexed ultrathin transistor arrays or organic transistors expand the ability of these surface arrays to record neuronal activity from the cerebral cortex from hundreds to thousands of channels with unmet spatiotemporal resolution.^[^ ^29^, ^30^, ^31^, ^32^ ^]^

Manufacturing on plastic thin foil permits high photolithography resolution patterning of the transducer (electrode or transistor) grids with spatial resolution as low as 10 µm. However, the relatively large Young's modulus (1–5 GPa) of thermoplastic polymers dictates thicknesses of a few micrometers only to enable sufficiently low bending stiffness and capillary forces high enough to conform to the convoluted surface of the brain.^[^ ^33^ ^]^ At this thickness scale, devices are harder to manipulate due to wrinkling and may be more prone to tear in some cases.^[^ ^34^ ^]^ Micro‐ECoGs on flexible foil technology are therefore often limited to be used over small surface areas, e.g., <1 cm wide strips, and/or their placement on the brain requires ad hoc deployment tools.^[^ ^20^, ^33^ ^]^ An alternative strategy that combines conformability and facile surgical handling is the use of soft polymers, with Young's moduli in the low MPa range, e.g., silicones, and sub‐millimetric thickness as carrier materials.^[^ ^35^, ^36^, ^37^, ^38^, ^39^, ^40^, ^41^, ^42^ ^]^

Here, we report on a silicone‐based ECoG that addresses the challenges of electrode customization and size, surgical handling and implantation, brain conformability and MRI compatibility. The soft grids are prepared using microfabrication to enable on‐demand and precise electrode size (250 µm to 5 mm in diameter) and layout, and high reproducibility. We validate surgical handling and clinical usability of the soft grids by implanting scaled prototypes in human cadaver specimens, both on standard brain areas and in more complex anatomical regions such as the lateral sulcus, a location currently rarely reached with clinical or flexible foil devices. We verify the compatibility of the soft ECoGs with 3T‐MRI clinical sequences, in gel phantom and human cadaver specimens, and against imaging artefact generation and local temperature increase. Finally, the recording and mapping capability of the soft ECoGs is verified by measuring somatosensory evoked potentials in minipigs in acute and subchronic settings, using different electrode size and pitch configurations, with low noise and high signal‐to‐noise ratio (SNR).

## 2 Conformability and Manufacturability of Soft Electrode Grids

**Figure 1:**
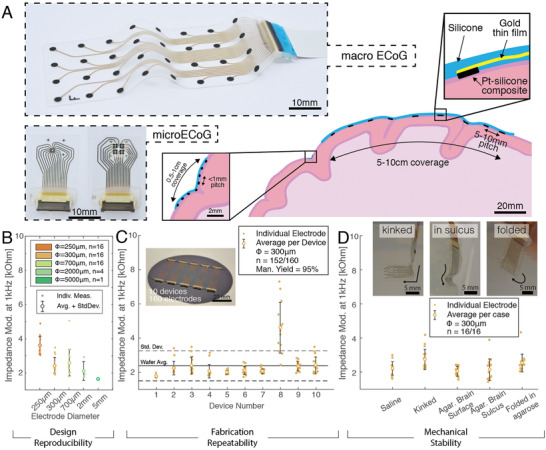
Technology validation of soft ECoG grids. A) Schematic representation of macro‐ECoG and micro‐ECoG (µECoG, in the middle inset) placement on the convoluted surface of the brain with corresponding length‐scales. The right inset shows the cross‐section of the device with the silicone substrate and encapsulation, the stretchable gold thin film and the Pt–silicone composite as electrode coating. Representative conformable grids from small (250 µm) to large (2.3 mm) diameter with small (2 × 2 mm^2^) to large (4 × 10 cm^2^) coverage are shown on the left. B) Modulus at 1 kHz of the electrochemical impedance spectroscopy measurements in saline solution of soft electrodes with different diameters as presented in (A). C) Manufacturing yield of the soft electrode arrays on a wafer scale illustrated by the impedance modulus at 1 kHz over 10 devices with 16 electrodes each. D) Robustness of the soft devices when handled. In different bending and folding scenarios (see inset), the impedance modulus at 1 kHz is reported to show the stability of the device when deformed mechanically.

Soft ECoGs (**Figure** **1**A) were manufactured following the silicone‐on‐silicon process described by Schiavone et al.^[^ ^40^ ^]^ with designs that comprise up to 32 channels and electrode diameter from 250 µm to 5 mm, laid out in different configurations as illustrated Figure 1A. Each ECoG layout, with the exception of the 250 µm diameter electrode one, is designed to cover an identical surface area, matching a 5 mm electrode diameter. Layout details are available in Figure S1 of the Supporting Information. The substrate and encapsulation are made of 75 µm thick PDMS membranes hosting 5 nm Cr/35 nm Au thin films stretchable interconnects. The electrode material is a dispersion of platinum (Pt) nanoparticles (ø = 0.2–0.4 µm) in a PDMS matrix. Individual devices are connected to custom‐made flexible circuit boards (see Figure S2, Supporting Information) using surface mounted device zero insertion force connectors. The fabrication process (illustrated in Figure S3, Supporting Information) enables manufacturing of ECoGs with millimeter to centimeter scale area coverage, limited here only by the available tooling (100 mm wafers in this work).

Bending stiffness (Equation (S1)) is the metric that determines the smallest cylinder radius a 2D sheet can conform to. Plastic foils need to be thinner than 10 µm (as the bending stiffness scales with the cube of the thickness and linearly with Young's modulus) to conform to curvatures of a few millimeters in radius, such as those found in the human brain.^[^ ^43^ ^]^ In the case of spherical or convoluted shapes such as the surface of the brain, bending stiffening will also constrain conforming to a second plane thereby hindering an intimate contact between the 2D sheet and the non‐gaussian surface over larger scales.^[^ ^44^ ^]^ Additionally, the handleability, intended here as the ease of using a device without dedicated tools, is severely affected by a reduced foil thickness and increased area. This is clearly illustrated in Figure S4D–F of the Supporting Information where different thicknesses of 4 cm × 8 cm polyimide foils (2.5 GPa Young's modulus) are laid on a brain gel model representing several orders of magnitude of bending stiffness (from Table S2, Supporting Information). On the other hand, while in the same bending stiffness range, elastomers such as PDMS are much softer and therefore allow conformability even at thicknesses in the 50 to 200 µm range, providing in turn more facile handling (Figure S4B,D, Supporting Information). By contrast, clinical ECoGs tend to spring back flat over curved surfaces (Figure S4A, Supporting Information). Conformability and handling are thus both critical aspects to account for in the development of large area (>1 cm^2^) subdural electrode arrays.

Soft electrode grids were characterized by electrochemical impedance spectroscopy in phosphate buffered saline (PBS) solution. The impedance modulus at 1 kHz (Figure 1B) benchmarks the fabrication process and performance of the soft electrodes of different diameters (the full spectrum is shown in Figure S5, Supporting Information). For stimulation, the electrode impedance should be kept to a minimum to enable voltage‐efficient current‐controlled stimulation pulses and ensure safe stimulation protocols.^[^ ^45^, ^46^ ^]^ For recording, although amplifiers are generally built for a broad spectrum of electrode impedance (this is not always the case with clinical amplifiers), low values reduce the thermal noise and ensure that the parasitic shunting between the electrode site and the amplifier is negligible.^[^ ^47^ ^]^ At 1 kHz, the impedance modulus of the soft electrodes (independently of the tested diameter) exhibits a near‐resistive phase and magnitude below 10 kΩ, dominated by the large sheet resistance of the microcracked gold film (5–10 Ω sq^−1^). This metric was selected as a benchmark parameter for process characterization. The manufacturing yield and process variability of the electrode grids were next assessed at the wafer level. A 16‐contact grid design was replicated ten times on a 4″ wafer (*n* = 160 electrodes, see inset of Figure 1C). On a representative wafer, the impedance at 1 kHz is 2.3 ± 0.8 kΩ with only 8/160 nonfunctional electrodes (defined as electrode exhibiting an impedance magnitude value above 10 kΩ at 1 kHz).

The functionality of the soft grids was tested in vitro under different conformability conditions. The grid was placed or inserted in an agarose gel brain model hydrated with PBS solution. When conforming to the gel or even completely folded over itself, the impedance at 1 kHz does not evidently differ from the in vitro measurements taken in a beaker, showing the mechanical robustness needed to endure the severe deformations occurring during surgery and use (Figure 1D), and consistent with previous reports on the mechanical reliability of this technology.^[^ ^40^ ^]^

## 3 Clinical Usability and MRI Compatibility

Most patients that today bear implanted electrodes in their body are not admitted to MRI scanners due to safety hazards. The electromagnetic field used for imaging can induce significant electromotive forces on the bulk metal electrodes due to magnetic induction,^[^ ^48^, ^49^ ^]^ and create local heating and temperature increases up to tens of degrees, due to coupling of RF‐fields with the metal contained in the implant acting as a receiving antenna. Current safety regulations limit the admitted temperature increase to 2 °C,^[^ ^50^ ^]^ and adverse effects can result in injury or even death of the patient. Beside safety, the materials used in clinical electrodes generate imaging artefacts due to the large difference in magnetic susceptibility between the metal and the surrounding tissue, hindering accurate diagnosis of the area of interest.^[^ ^51^, ^52^ ^]^

**Figure 2:**
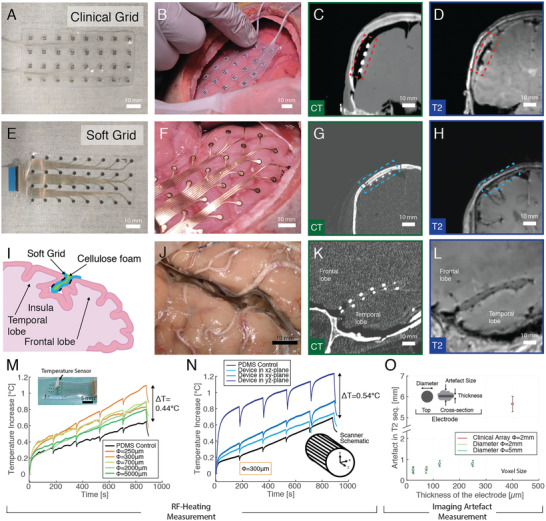
MRI compatibility and study in human cadaveric models of large‐scale soft ECoG grids. A) Clinical ECoG grid. B) Photograph of the placement of the clinical ECoG grid on the surface of the cadaveric specimen. C,D), Extracted image of the implanted clinical device in CT and T2‐weighted MRI scans, respectively. The red box indicates the position of the implanted device. E) Soft ECoG grid. F) Photograph of the placement of the soft ECoG grid on the surface of the cadaveric specimen. G,H) Extracted image of the implanted soft device in CT and T2‐weighted MRI scans, respectively. The blue box indicates the position of the implanted device. I) Implantation schematic of the soft ECoG grid in the Sylvian fold. J) Image of the placement of a folded soft ECoG grid in the Sylvian fold of the cadaveric specimen. K,L) Extracted image of the implanted clinical device in the Sylvian fold by CT and T2‐weighted MRI scans, respectively. The image shows the location of the electrode array in the Sylvian fold along the length of the device. M) Monitoring of the RF‐heating at the surface of the electrode during a T2‐weighted turbo‐spin echo MRI sequence in a 3T clinical scanner for different electrode diameters (colored lines) compared to control (in black). N) Monitoring of the RF‐heating at the surface of the electrode during a T2‐weighted turbo‐spin echo MRI sequence in a 3T clinical scanner for an electrode diameter of 300 µm when positioned in different imaging planes (blue colors) compared to control (in black). O) Measurement of the out‐of‐plane imaging artifact of a T2‐weighted MRI sequence of soft electrodes with different diameters (green colors) and thicknesses compared to a clinical electrode array (in red).

In this context, we tested the hypothesis that the soft ECoG embedded in silicone and engineered with a thin‐film conductor technology would offer the inherent advantage of poorly interacting with external oscillating electromagnetic fields such as those used for MRI. We verified the usability of soft ECoGs in the clinical scenario of implantation and imaging in a human cadaver specimen. A soft electrode grid mirroring the design of a clinical 32‐channel ECoG used for epilepsy monitoring was produced. A clinical grid (**Figure** **2**A) was placed on the cortex and its mechanical signature required the surgeon to push the electrodes against the surface of the brain to establish electrical contact on all the electrodes, as shown Figure 2B. Poor electrical contact was detected by means of impedance measurements, showing open‐circuit in the corner electrodes (Figure S6A, Supporting Information). On the other hand, the soft electrode grid (Figure 2E), whilst easily handled, conformed to the brain surface by capillarity thanks to the low modulus of silicone (**Figure** **2F**), and enabled intimate, low‐impedance contact (Figure S6B, Supporting Information).

**Figure 3:**
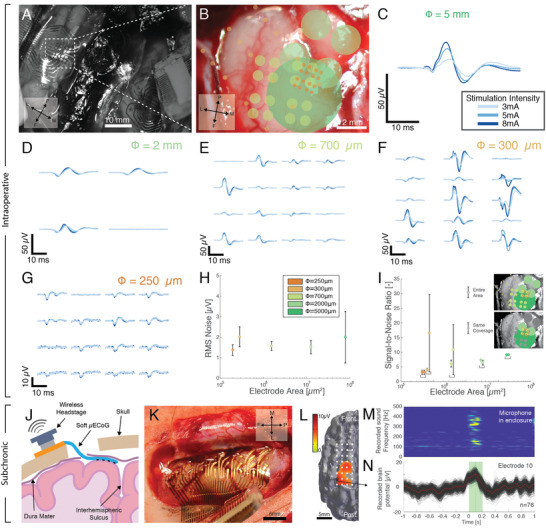
In vivo soft µECoG recordings in minipigs in acute and chronic settings. A) Overview of the placement of the conformable electrode arrays on the motor cortex on the left and right hemisphere. B) Reconstruction of the sequential position of the electrodes when recording with the different grid layouts. C–G) Stimulus‐locked somatosensory evoked potential recordings on the cortex from snout stimulation with the 5 mm, 2 mm, 700 µm, 300 µm, and 250 µm electrode diameter electrodes, respectively. The reported signal is shown from 5 ms after the stimulation and up to 100 ms after the stimulation; examples of raw traces can be found in Figures S19–S23 of the Supporting Information. H) Baseline noise recordings with the soft electrodes depending on their diameter, examples of raw traces can be found in Figures S24–S28 of the Supporting Information. I) Signal‐over‐noise ratio (SNR) of the recorded SSEPs with the soft electrode grids. Circles (●) indicate the SNR when comparing all electrodes from the device and diamonds (◆) indicate the SNR when comparing only selected electrodes covering the same selected region. The inset illustrates which electrodes are considered in each case. J) Schematic representation of the surgical approach for the subdural recording with the soft µECoG. K) Intraoperative picture of the electrode array placed subdurally over the left prefrontal cortex. L) Activation map of the cortical activity elicited by vocalizations in this minipig at a latency of 100 ms after vocal onset. M) Spectrogram of a single vocalization recorded by a microphone. N) Evoked potentials recorded by an electrode over the sensorimotor cortex during a freely moving recording session (red: average potential over *n* = 76 vocalizations; black = 1000 bootstrap averages). The averaged vocal production time is represented by the green rectangle and the vertical green line indicates the time of the map in (M).

Next, both devices were imaged in situ with a clinical computed‐tomography (CT) scanner and a Siemens 3T clinical MRI scanner. Both devices are visible on the CT scans (Figure 2C,G), easily enabling localization of the electrodes on the tissue. From the MRI image, especially on the T2‐weighted sequence (Figure 2D; T1‐weighted sequence in Figure S7A, Supporting Information), we observed the clinical grid creates image artefacts of nearly 1 cm length, shadowing the gray matter in the surrounding region. On the other hand, the soft array was imperceptible on the T2‐sequence enabling crisp imaging of the tissue all around the implanted grid (Figure 2H; T1‐weighted sequence in Figure S7B, Supporting Information).

In epilepsy patients, a large number of seizure foci are located on the temporal lobe, and in some more rare cases on the insula, a cortical region situated at the bottom of the lateral sulcus (Figure 2I), which separates the frontal from the temporal lobe. Clinical grids being too thick and rigid, cannot access this area. We explored if the soft ECoGs would instead enable electrophysiological access to this deep brain area. After accessing the lateral sulcus, a soft grid was folded along its length then inserted in the fold as shown in Figure 2J. The corresponding CT scan shows the lateral sulcus along its length and cross‐section, respectively, where the electrodes are clearly visualized in the sulcus (Figure 2K). MRI images from the T2‐sequence show even more clearly the electrodes in contact with the frontal lobe and the temporal lobe from within the lateral sulcus (Figure 2L; perpendicular view in Figure S8A,B, Supporting Information). Each individual electrode is visible as a ≈0.5 mm wide artefact that is large enough to locate the electrode but small enough to visualize the brain tissue. This experiment demonstrates that by carefully designing the electrode layout, a soft ECoG could enable mapping of both lobes as well as the insula at the bottom of the sulcus. The location of each electrode would then be tracked relative to anatomical landmarks by MRI.

Next, we verified that the soft grid does not produce unsafe heating during MRI scanning. The soft implants were placed on an imaging phantom and then scanned. In a 15 min long T2‐weighted turbo spin echo sequence, which uses the largest RF‐field gradients thereby leading to the largest heating,^[^ ^50^ ^]^ the maximum temperature increase (compared to plain PDMS control) measured was around 0.44 °C for the smallest electrodes (*⌀* = 250 µm, Figure 2M), and 0.54 °C when positioning the device in different planes of the scanner (Figure 2N). The increase of temperature in the control reflects the phantom and temperature probe.

To evaluate the imaging artefact, similar electrode geometries were compared: *ø* = 2 mm and 5 mm, with different thicknesses (25 µm, 75 µm, 125 µm, and 250 µm) for the soft grids, and *ø* = 2.3 mm and 400 µm thickness for the clinical device. The imaging results show that the dark‐contrasted artefact generated in a T2‐weighted sequence of maximum 0.79 ± 0.14 mm in the out‐of‐plane direction for the soft grids, while the clinical grids create artefacts of around 5.6 ± 0.36 mm (Figure 2O; Figure S9A,B, Supporting Information). In the soft grids, the magnetic susceptibility mismatch at the origin of artefacts is mitigated by the small volume of metal required to form interconnects and electrodes, as opposed to what observed with clinical devices.

## 4 Subdural Surface Recordings in Minipig Models

The recording capabilities of the soft grids were investigated both in pig and minipig models (*n* = 2 animals). We selected these translational models for their large and convoluted cortical brain surface.

In an acute setting and under anesthesia, we percutaneously stimulated the animal snout with bipolar needle electrodes at different locations (overview in Figure S10, Supporting Information). Somatosensory evoked potentials (SSEPs) lead local field potentials (LFPs), which were recorded with the soft electrode arrays placed on the cortex as shown Figure 3A.^[^ ^13^ ^]^ Soft grids with different electrode layouts were positioned one after the other over the same cortical region, recording LFP responses to snout stimulation. Contralateral placement maps (extracted from surgery images and aligned with anatomical landmarks) of the different grid designs are presented Figure 3B for one of the animals in the study. The recorded signals collected by the grids with increasing electrode diameter and for the same snout stimulation are presented Figure 3C–G (and across two animals in Figure S12, Supporting Information). All electrode designs enabled recording of SSEPs of amplitude ranging from 10 to 100 µV. For all designs, increasing the stimulation amplitude increased the peak amplitude of the evoked response, confirming that recorded signals originated from the sensory system. The time delay between the stimulation (*t* = 0 ms) and the first peak (around *t* = 15 ms) was consistent with SSEPs recorded in intraoperative neuromonitoring in humans (examples of raw traces recorded across all electrode diameters are shown in Figures S19–S23, Supporting Information). Additionally, when mapping the different locations of the snout, the cortical activation map changed as illustrated in Figure S11 of the Supporting Information, indicating that separate channels on the grids can resolve different cortical events.

Figure 3C–G highlights several key observations. Location‐dependent patterns of the recorded activity were only visible in the maps obtained with intermediate size electrodes. For electrodes of 2 mm, 5 mm or 250 µm diameter, the recorded signals were essentially identical across the grid, showing no evident difference in both amplitude and timing. With reference to the placement maps (Figure 3E), we hypothesized that the bigger electrode diameters (2 and 5 mm) did not capture crucial submillimeter scale variations that appeared with the other electrode diameters. Conversely, electrodes of the smallest diameter (250 µm) failed to pick up topological differences in cortical signals because the inter‐electrode pitch (350 µm) was too small to differentiate populations of neurons involved in different somatosensory circuits.

Noise levels and signal amplitude are crucial metrics, especially for applications such as brain–machine interfaces that rely on accurate and precise neural signal detection to drive a prosthesis. For each tested soft grid, we quantified their root‐mean‐square noise level, *n*_rms_, and SNR characteristics (the baseline activity is shown in Figures S24–S28, Supporting Information). Figure 3H shows a comparison of the extracted *n*_rms_ across electrode diameters, showing a low noise level around 2 µV with no size dependency. Figure 3I plots the average SNR per grid and as a function of electrode diameter when the soft grids were probing approximately the same cortical area (as represented by the colored electrodes in the inset of SNR was typically 5–10. When comparing all electrodes performance, the 300 µm diameter electrode grid with the biggest surface coverage showed outliers in SNR (up to 30), possibly originating from signals outside the covered region of the other devices.

Our results indicate that in the context of minipig somatosensory cortex recording, soft grids with electrodes of a few hundred micrometers (e.g., 300 µm) diameter with submillimeter pitch (e.g., 700 µm) should provide sufficient spatiotemporal resolution to avoid signal redundancy. Albeit promising, these preliminary results require further corroboration with electrode placement with greater position accuracy and further signal analysis. The data shown here illustrate the capability of the soft ECoG technology to rapidly create various electrode layouts that will enable further investigations of neuroengineering and neuroscientific questions.

Finally, we conducted a pilot experiment designing a 32‐contact (4 × 8 grid) soft grid (Figure S13, Supporting Information) p to attempt subchronic recording of vocalization‐elicited patterns in a minipig model. The soft grid was surgically positioned above the motor cortex (identified by prior MRI and CT‐scanning). Following durotomy, the grid (4 × 15 mm^2^) was partially pushed below to the skull to reach the cortical region of interest while minimizing the size of the craniotomy (Figure 3J,K). Two weeks postimplantation, we recorded the animal's spontaneous vocalization through a microphone in its enclosure and time‐locked cortical signals acquired from a wireless headstage connected to the implant. Figure 3K shows a confined distribution of the measured cortical activation over the posterior part of the array enabled by the large coverage of the implant on the brain. An example vocalization pattern and the evoked potentials are shown Figure 3M,N, thereby demonstrating reproducible motor responses evoked by vocalizations.

## 5 Conclusion

In this study, we report a fully customizable silicone technology platform enabling the fabrication of MRI‐compatible, multichannel, and soft electrode grids that facilitate the interfacing of large and convoluted brain areas. The design and size versatility of the technology will enable cortical mapping with higher spatial resolution and access to complex surgical “terrains” such as those encountered in the case of brain resections in tumor or epilepsy cases.

Within established clinical procedures, the ability to better define intervention areas on the cortex and adapt to the reality of the case will offer improved clinical outcomes after resection surgeries. Beyond current practice, soft grids will enable interfacing with previously inaccessible anatomical targets such as the Sylvian fold as presented herein but also, by analogy of the methods, the interhemispheric sulcus^[^ ^53^ ^]^ and others regions. Soft technology can be leveraged to perform mapping operations (for recording as presented here or stimulation as presented in refs. ^[^ ^40^ ^]^ and ^[^ ^54^ ^]^) in such areas with high‐resolution. MRI‐compatibility will enable safe and precise localization of the electrodes within the tissue, with the added advantage of allowing diagnostic structural and functional imaging of the surrounding tissue, currently impossible with clinical grids. The rapid, inexpensive, and scalable manufacturability of soft ECoG grids may in the future unlock the opportunity to produce targeted devices designed based on patient‐specific anatomy and surgical needs.

Further translation of the soft grid neurotechnology will require impelling advances in system integration, especially on durable and reliable industry‐standard electrical leads and connectors^[^ ^55^ ^]^ to be fed through the skull. The mechanical compliance of the soft grids calls for radically different designs that must accommodate the heterogeneous electromechanical properties of the different components of the full system. Advances will be further required in increasing the density of recording electrodes, a metric that today is driven by a marked trade‐off between mechanical compliance of the materials used and the associated manufacturing capabilities.^[^ ^18^ ^]^

We finally anticipate that clinical approval of such soft neurotechnology will open up to new diagnostic or therapeutic avenues such as brain–machine interfaces for restoration of motor,^[^ ^8^ ^]^ language^[^ ^56^ ^]^ or other neural functions that are presently limited or unexplored due to current technological limitations.

## Conflict of Interest

FF., G.S., N.V., L.S., S.P.L., G.C., and J.B. hold various patents in relation with the present work. S.PL., G.C., and J.B. are cofounders of GTXmedical. FF., N.V., L.S., and S.P.L. are cofounders of Neurosoft Bioelectronics SA.

## Supporting information

Supporting Information

## Data Availability

Data available on request from the authors.
