# Supplementary material for: MRI‐Compatible and Conformal Electrocorticography Grids for Translational Research
Source: Adv Sci (Weinh). 2021 Mar 8;8(9):2003761. doi: 10.1002/advs.202003761 (PMC8097365; doi:10.1002/advs.202003761)
Supplement: Supplementary file 1 — Supporting Information [file ADVS-8-2003761-s001.pdf]

© 2021 Wiley-VCH GmbH

ADVANCED SCIENCE
Open Access

Supporting Information

for *Adv. Sci.,* DOI: 10.1002/advs.202003761

MRI-compatible and conformal electrocorticography grids for translational research

*Florian Fallegger[1], Giuseppe Schiavone[1], Elvira Pirondini[2,3†], Fabien B. Wagner[3,4†], Nicolas Vachicouras[1], Ludovic Serex[1], Gregory Zegarek[5], Adrien May[5], Paul Constanthin[5], Marie Palma[6], Mehrdad Khoshnevis[6], Dirk Van Roost[5,7], Blaise Yvert[6], Grégoire Courtine[3,4], Karl Schaller[5†], Jocelyne Bloch[2,3†], Stéphanie P. Lacour[1]**

((Supporting Information can be included here using this template))

© 2021 Wiley-VCH GmbH

Supporting Information

MRI-compatible and conformal electrocorticography grids for translational research

*Florian Fallegger[1], Giuseppe Schiavone[1], Elvira Pirondini[2,3†], Fabien B. Wagner[3,4†], Nicolas Vachicouras[1], Ludovic Serex[1], Gregory Zegarek[5], Adrien May[5], Paul Constanthin[5], Marie Palma[6], Mehrdad Khoshnevis[6], Dirk Van Roost[5,7], Blaise Yvert[6], Grégoire Courtine[3,4], Karl Schaller[5†], Jocelyne Bloch[2,3†], Stéphanie P. Lacour[1]**

Methods

Microfabrication

The conformable µECoGs are fabricated using processes borrowed from standard microfabrication steps and soft lithography. The process flow is illustrated in detail in Supplementary Figure 3. In short, on a 4inch silicon carrier wafer, a 1µm thick dextran layer (70kDa, Arcos Organics) is spin-coated by using a 20%wt solution in deionized water then dehydrated at 150°C for 1min on a hotplate. Then a 75µm thick silicone layer (Sylgard 184, Dow Corning) is spin coated at 750rpm and cured at 75°C for 2hours in an oven. A 23µm PET sheet (Mylar, Lohmann Technologies) is laminated on top of the silicone and then is micromachined by an excimer femtosecond laser (Optec) to form the shadow mask. The tracks are removed at once using tape. The metallic layer (5nm Cr and 35nm Au) is thermally evaporated under vacuum (base pressure <2·10-6 mbar, Auto 306, Edwards) as previously reported. In parallel, the encapsulation, formed by a triple layer of 23µm PET/75µm PDMS/23µm PET (as described above), is machined by fs laser to define electrode via and contact pad. After removal of a PET layer on both, the encapsulation and the substrate are O2 plasma activated (Diener plasma) then aligned and bonded on a wafer level using alignment

crosses. The electrode sites are covered by screen-printing with a dispersion made of platinum nanoparticles (ø=0.27-0.47µm, Strem Chemicals Inc.) and PDMS at a weight ratio of 70%, using cyclohexane (Sigma Aldrich) to help mixing. The mixture is degassed and then cured at 55°C for 4 hours. To connect to the device, an uncured silver epoxy paste (Epotek H27D) is used as a medium between the stretchable gold thin film and a surface mounted zero insertion force device (Hirose) used as connector, connected then to a custom-made polyimide-copper flexible printed circuit board, over molded with silicone adhesive (One component silicone sealant 734 clear, Dow Corning). The device outline is defined by fs laser machining with subsequent release in deionized water. The brain phantom is made by dissolving 3%wt agarose (Electran) in phosphate buffered solution (Gibco PBS, pH 7.4, 1X) in a mold.

Testing

The electrodes are characterized by electrochemical impedance spectroscopy in a 3-electrode configuration (Gamry Instruments Reference 600 Potentiostat) in saline solution (Gibco PBS, pH 7.4, 1X) at room temperature with a platinum wire counter and Ag/AgCl (Metrohm, El. Ag/AgCl DJ RN SC: KCl) reference electrode. The frequency is swept from 1MHz to 1Hz, using a 100mV excitation signal.

Conformability testing on mock-brain

PDMS (Sylgard 184, Dow Corning, including a blue silicone dye (Smooth-On, sil pig blue)) thin films were fabricated as described in the microfrabrication section above, varying the spin-coating speed to achieve different thicknesses. Polyimide (Dupont, PI 2611) thin films were prepared by spin-coating the liquid monomer onto silicon wafers and baking the layer according to the manufacturer’s instructions, the different thicknesses were achieved by varying the spin-coating speed. Mock-brains were prepared by dissolving agarose into PBS (1%wt) and filling a silicone mold created by negative molding of a 3D printed human brain hemisphere. Once the mock-brain was demolded, the surface was wetted, then the different layers (PDMS and PI) were laid sequentially onto the surface and wetted with saline again. Pictures were acquired with a handheld camera.

Cadaver head and MRI

In a human cadaver specimen, a large craniotomy is made over the temporal lobe exposing the lateral sulcus, the frontal and the temporal lobes. The dura mater was carefully cut and a

flap was opened to expose the cortex. The electrode grid is placed on the surface of the brain. Saline is applied to the brain to keep it hydrated and ensure optimal capillary action between the electrode array and the brain surface. The grid is then covered again by the dura mater, bone flap and skin. The cadaver specimen is imaged using a clinical computed-tomography (CT) scanner (GE 750 HD CT, General Electric USA, 120-kVp tube voltage, 22.9 effective mAs tube current without current modulation, pitch of 1.375, slice thickness of 0.625, and table speed of 55 mm/rotation) and a 3T MRI scanner (Siemens Magnetom TrioTim 3T, sequence parameters shown in Supplementary Figures Supplementary Figure 14, 15). The images are exported and processed using a DICOM viewer (Miele-LXIV). For the lateral sulcus approach, the cortex is exposed of the sulcus and the blood vessels are carefully separated between the two lobes. The soft electrode array is inserted by first folding it in half. A cellulose gel is inserted over the array to fill the void above the device. The dura mater and bone flap are closed again together with the skin before imaging.

MRI phantom measurements

The imaging phantom is prepared by dissolving 3%wt agarose (Electran) in phosphate buffered solution (Gibco PBS, pH 7.4, 1X) in a mold. For imaging, the device under test (Soft electrode or ADTech ECoG Grid) is placed between two slabs of agarose in a glass container filled with PBS. The container is placed in the head coil of a Siemens 3T MRI scanner (Siemens Magnetom Prisma 3T). Different sequences are run to test the imaging compatibility (details of the sequence in Supplementary Figure 16**Error! Reference source not found.**, 17). The images are exported using a DICOM viewer (Miele-LXIV) without further processing except adjusting for the position, and adjusting brightness and contrast. For the heating measurements, the device under test is placed on top of a phantom a thermocouple is placed over the electrode sites and the system is covered by insulating foam to ensure close contact between the electrodes and the temperature probe. A T1-weighted turbo spin echo sequence (parameters in Supplementary Figure *18*) is run for 15 min split in 3min sequences and the temperature is measured continuously using an ADC (0.1 Hz acquisition frequency). The device is either placed in the axis of the scanner or in orthogonal directions to it.

Acute minipig experiment

Göttingen minipig or young farm pigs (n = 2) were used in the acute in vivo recording experiment. First, each animal was sedated with an intramuscular ketamine injection,

followed by isoflurane delivered with a face mask. Once the animal was fully anesthetized, an intravenous catheter was placed in the ear vein. A mix of propofol and saline solution was administrated as anesthetic during the surgery. The animal was taken off the face mask and intubated with active respiration. Heartrate, body temperature and blood oxygen pressure were monitored continuously. The animal was placed on the surgery table (over a heating blanket) covered with sterile drapes with top of the head exposed. A large frontal to posterior incision over the skull was performed. The skin and underlying muscles were separated from the skull and pulled aside with forceps, to expose as much skull area as possible. Four burr holes were drilled with a 5 mm diameter drill at the corners of the exposed skull. The skull between the holes was drilled to open a square bone flap. The bone flap was removed to expose the dura. The dura mater was cut open with a scalpel blade and flapped over until 2mm lateral from the midline. The brain was continuously kept wet with saline solution. The arrays were placed over the defined cortex region by holding the fPCB cable with a custom-made 3D printed frame. The snout was stimulated with bipolar needle electrodes (Ambu Neuroline Twisted Pair Electrodes 745 12-100, Ambu) (3, 5 and 8 mA amplitude, cathodic-first biphasic pulses, 1 Hz repetition rate, 300 µs pulse width) using an external pulse generator (AM System). Evoked potentials were recorded differentially between the electrode site and a subdural ground wire on the frontal end of the craniotomy (triggered averaged recording (n~60), band-pass filtered 1-5000 Hz, notch filter at 50 Hz and harmonics) using a commercially available amplifier and data acquisition system (PZ5 preamplifier and RZ2 base station, Tucker-Davis Technologies).

Chronic minipig experiment

An Aachener minipig was used in the subchronic in vivo recording experiment. The animal was anesthetized with isoflurane with a face mask. Once the animal was fully anesthetized, an intravenous lead was placed on the ear. Heartrate, body temperature and blood oxygen pressure were monitored continuously. The animal was placed on the surgery table (over a heating blanket) covered with sterile drapes with top of the head exposed. A large frontal to posterior incision over the skull was performed. The skin and underlying tissue were separated from the skull on an area the size of the chronic titanium chamber housing the connector. A cranial window was opened with a bone drill. The bone flap was removed to expose the dura. The dura mater was cut open with a scalpel blade and flapped over. The brain was continuously kept wet with saline solution. The soft electrode array was placed on

the brain and gently pushed under the skull. After fixing a reference wire under the dura and a ground wire on the skull, the dura was closed again and covered with artificial dura mater. The bone flap was placed back and secured with titanium pieces onto the skull. A titanium chamber was screwed into the skull using screws and the inside of the chamber was filled with dental cement securing the connector in place. An acrylic cap was screwed over the chamber to protect the connector during the movement of the animal. After two weeks of post-operative recovery, the animal was placed into a soundproof chamber equipped with a microphone. A wireless amplifier headstage (Multichannel systems GmbH) was plugged into the connector on the head of the animal to measure the brain activity. Both signals were acquired synchronously. The brain signals were acquired at 20 kHz, low-pass-filtered below 10 Hz and down-sampled at 200 Hz. The brain recording was averaged across 76 spontaneous vocalizations triggered by their onset time. The interval during which the averaged evoked response exceeded the baseline noise level was isolated according to a Welch test ensuring $P<0.05$ statistical significance. Spatial activity maps projected onto the cortical anatomy were thresholded according to this significance level.

Supplementary Figures

Supplementary Table 1 Comparative overview of clinical and research electrodes for recordings of the brain

Electrode Type / Parameter	ADTech Corp. and PMT Corp.	High-density research electrodes (ADTech Corp.)	Cortec	NeuroOne	Neurogrid	This work
Reference	Company website	[1]	Company website	Company website	[2,3]	-
Encapsulation material	High shore silicone	High shore silicone	High shore silicone	Polyimide	Parylene	Low shore silicone
Interconnect type	Individual wires	Individual wires	Patterned 25µm-thick PtIr sheet	Platinum thin film (100nm)	Pt/Au thin film (150/50nm)	Gold thin film (35nm)
Electrode site material	Bulk PtIr 0.4 mm thick	Bulk PtIr 0.4 mm thick	Patterned 25µm-thick PtIr sheet	Platinum thin film (100nm)	PEDOT:PSS	Pt nanoparticle in silicone matrix
Device Thickness	0.5 - 1 mm	0.5 - 1 mm	0.75 mm	80 µm	4 µm	150 µm
No. of channels	2 - 64	256	4 - 64	2 - 16	240	1 - 32
Recording Area min.	1 x 2 cm^2	-	1 x 4 cm^2	1 x 2 cm^2	0.1 x 0.1 cm^2 in [2]	0.2 x 0.2 cm^2
Recording Area max.	7 cm x 7cm	6.4 x 6.4 cm^2	7 cm x 7cm	3 cm x 3 cm	1.4 x 1.4 cm^2 in [3]	3 x 7 cm^2
Electrode Pitch	10 mm	4 mm	10 mm	10 mm	20 µm in [2]<br>2 mm [3]	0.2 - 10 mm
Electrode Diameter	2.3 mm	1.17 mm	2.7 mm	3 mm	10 µm	0.25 mm – 5 mm
Clinically available	US: Yes<br>EU: Yes	US: No<br>EU: No	US: Yes<br>EU: No	US: Yes<br>EU: No	US: No<br>EU: No	US: No<br>EU: No
Demonstrated or permitted implantation period in humans	Intra-operative Subchronic	Intra-operative Subchronic	Intra-operative Subchronic	Intra-operative Subchronic	Intra-operative	-

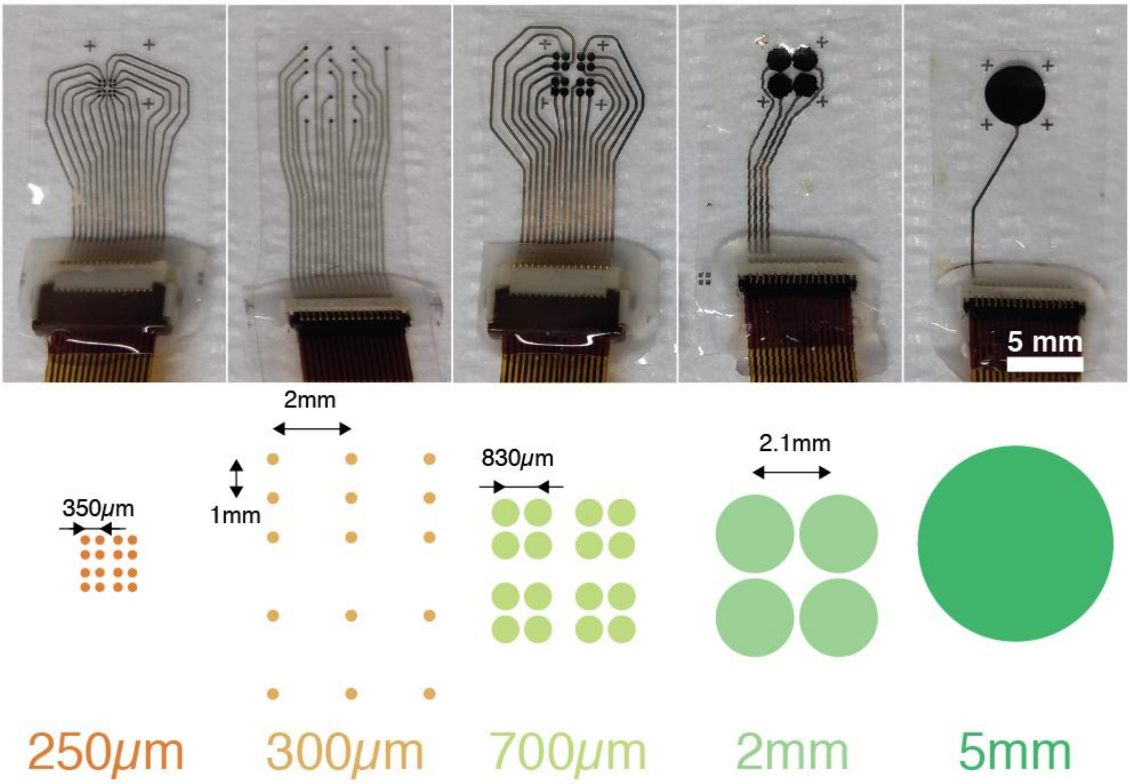

Supplementary Figure 1 Overview of the electrode layouts for the different conformable electrode arrays.

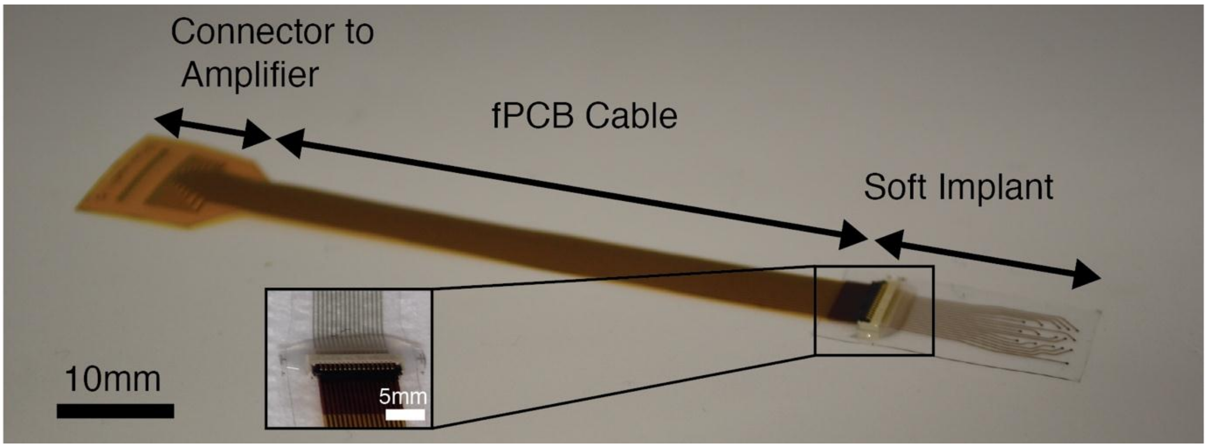

Supplementary Figure 2. System overview to connect the soft electrode array to the amplifier for recording brain activity. The inset shows the transition from the elastomeric substrate to the flexible printed circuit board by the mean of a surface mounted zero-insertion force connector.

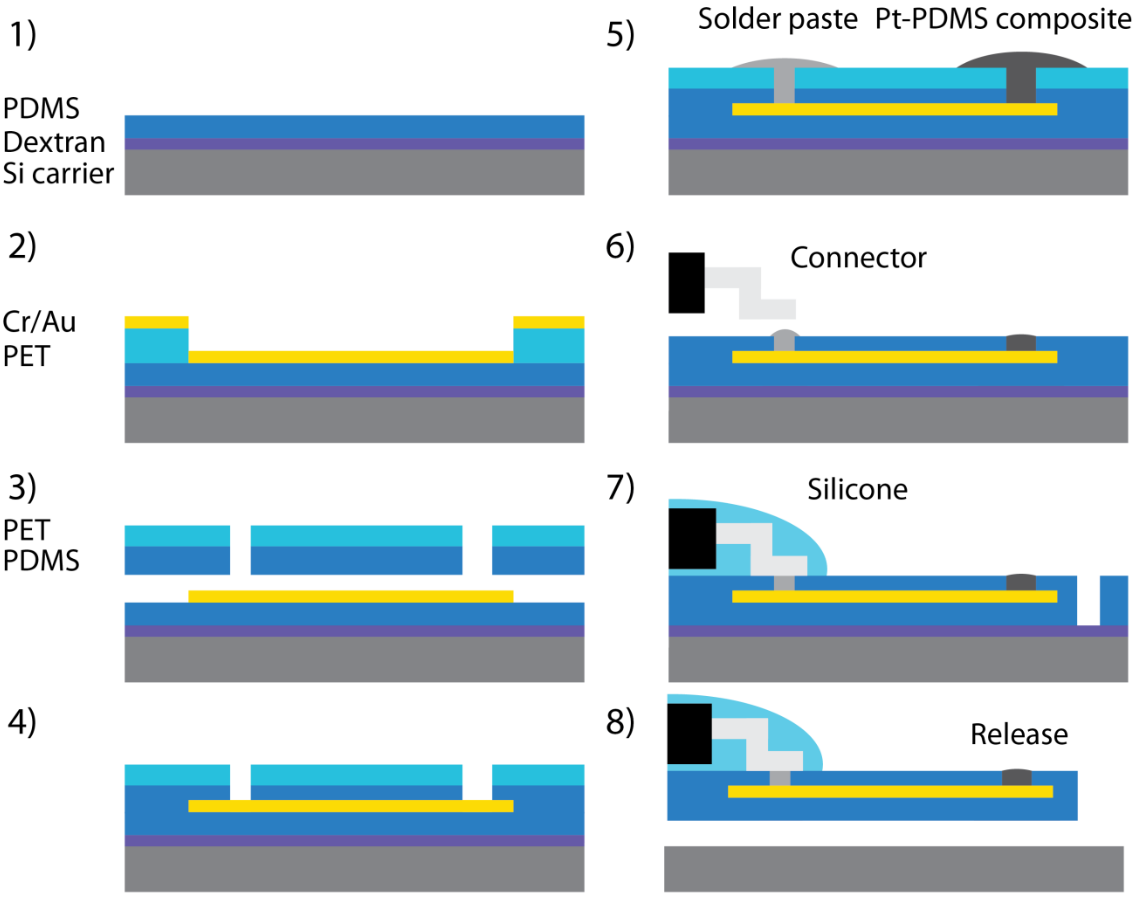

Supplementary Figure 3. Process flow for the fabrication of the conformable ECoG devices. **1)** Substrate preparation by subsequent spin coating of a dextran release layer and PDMS onto a silicon carrier wafer. **2)** Evaporation of the stretchable gold interconnects through a PET stencil mask. **3)** Alignment of the PDMS/PET encapsulation onto the interconnects. **4)** Interconnects encapsulated between the two silicone layers. **5)** Screen-printing of the platinum-silicone dispersion over the electrode sites and solder paste onto the connection pads through the PET stencil mask. **6)** Placement of the surface mounted connector. **7)** Encapsulation of the connector with silicone sealant and cutting of the implant shape. **8)** Release of the device from the carrier wafer.

Supplementary equation:

Bending stiffness:

$D = \frac{Eh^3}{12(1-\nu^2)}$ (1)

With D the bending stiffness in [Pa·m^3], E the Young's modulus of the material in [Pa], h the thickness of the layer in [m] and ν the Poisson ratio in [-].

Supplementary Table 2. Materials properties used for the calculation of the bending stiffness. The value for PDMS is extracted from [4] and for polyimide from [5].

Material	Young's Modulus [Pa]	Poisson ratio [-]
Polydimethylsiloxane (Sylgard 184)	$1.5 \cdot 10^6$	0.499
Polyimide (Dupont Kapton)	$2.5 \cdot 10^9$	0.34

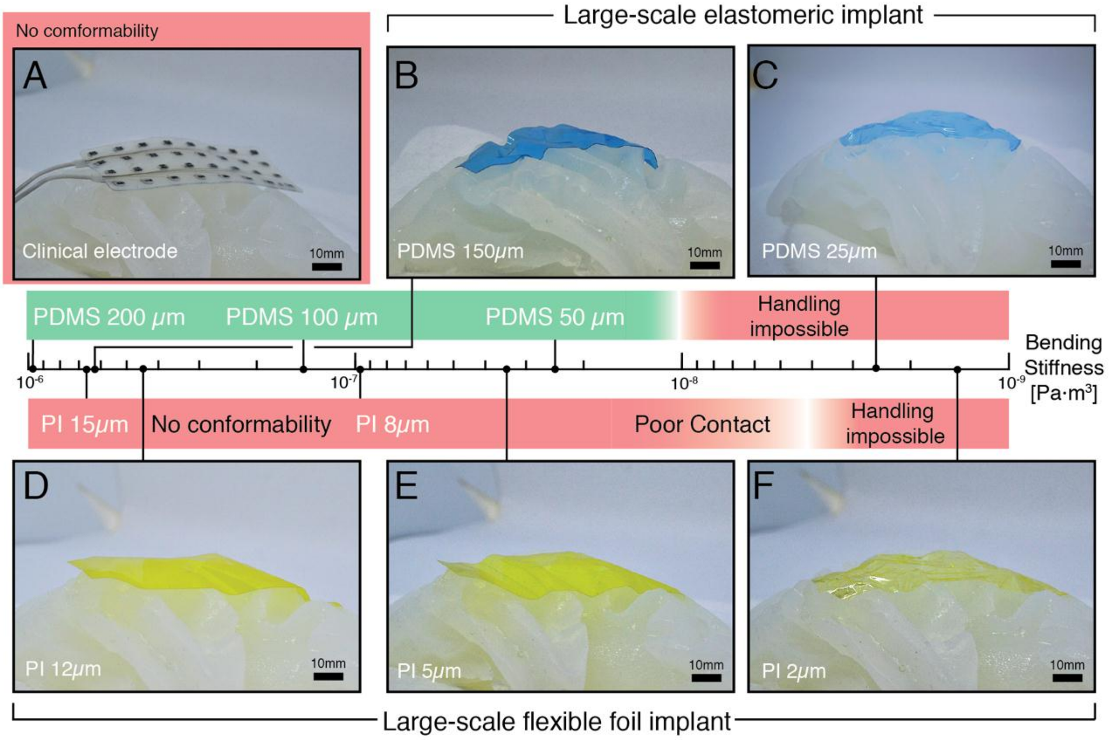

Supplementary Figure 4 The conformability of different mock-up devices (size of 4 x 10 cm^2) is studied on an agarose gel brain model with the corresponding bending stiffness reported on the middle scalebar, along with a qualitative evaluation of the conformability and handleability. Tested materials: **A)** Clinical ECoG array. **B), C)** Silicone membrane. **D), E), F)** Polyimide foil.

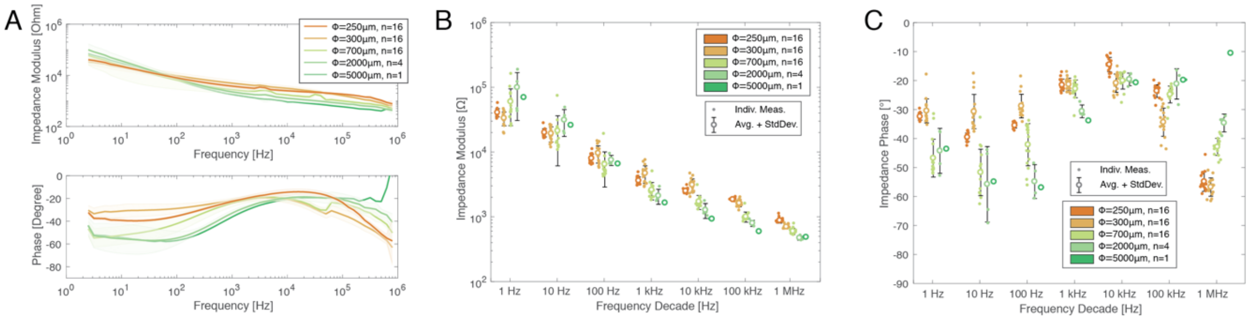

Supplementary Figure 5 Full spectrum of the impedance and phase of electrochemical impedance spectroscopy measurements in saline solution of soft electrodes with different diameters. **A)** Average and standard deviation per diameter of the impedance modulus (top) and phase (bottom) spectrum. **B), C)** Boxplot of the impedance modulus (B) and phase (C) per diameter type at each frequency decade.

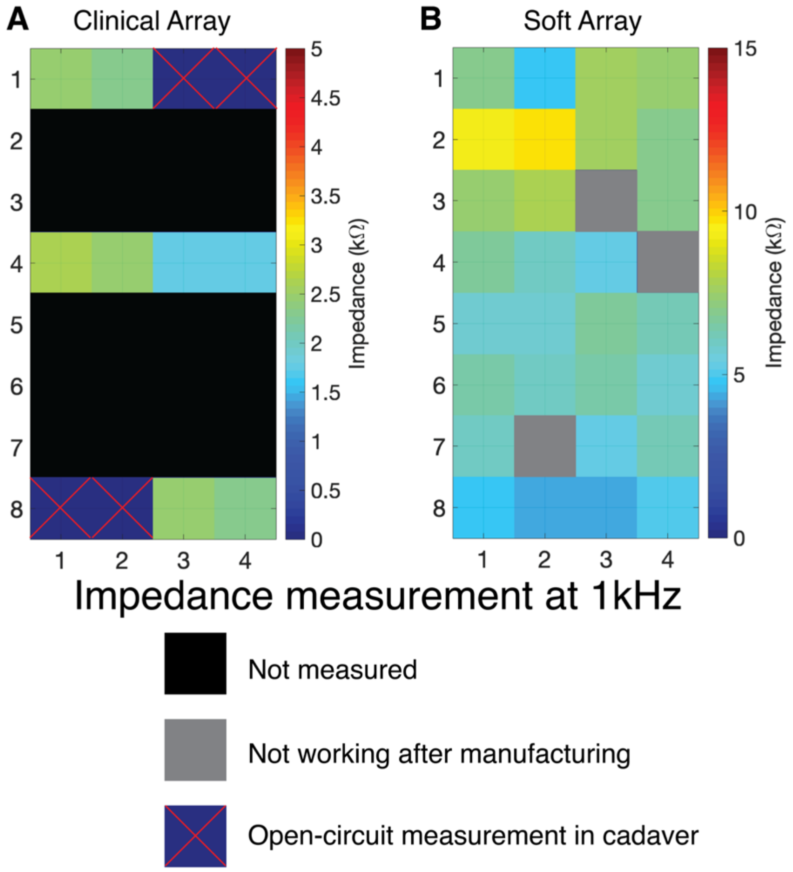

Supplementary Figure 6. **A)** Colormap of the measurement of impedance modulus at 1kHz of the clinical ECoG grid implanted in the cadaveric specimen versus a platinum counter-electrode. **B)** Colormap of the measurement of impedance modulus at 1kHz of the soft ECoG grid implanted in the cadaveric specimen versus a platinum counter-electrode. In A) and B), a black color indicates that the electrode was not measured due to time constraints, grey indicates that the electrode was not working post-fabrication and blue with red cross indicates an open-circuit value of the impedance, indicating no contact between the tissue and the electrode.

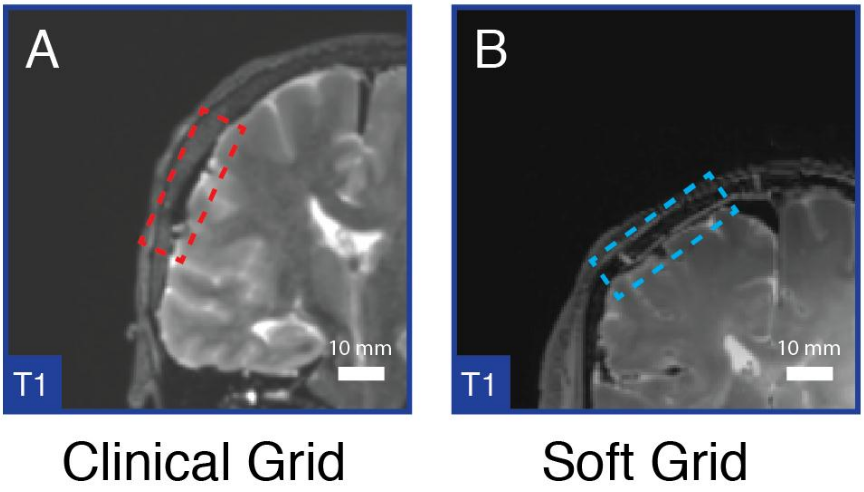

Supplementary Figure 7. Extracted coronal images of the implanted **A)** clinical device and **B)** soft grid in T1-weighted MRI scans, respectively. The box indicates the position of the implanted device.

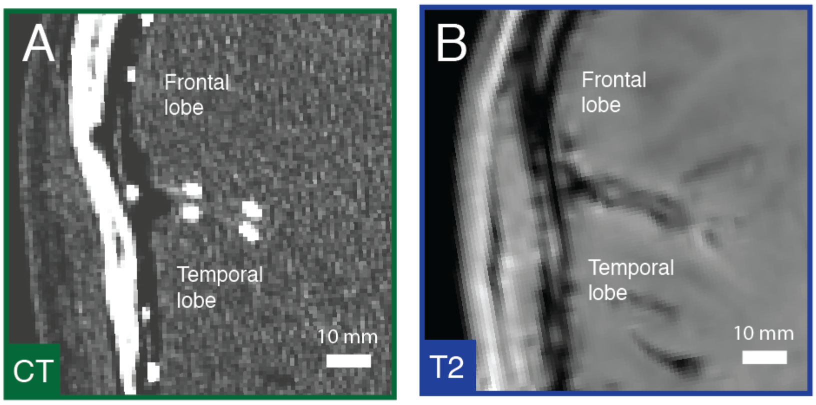

Supplementary Figure 8. Extracted coronal images of the implanted soft grid in the lateral sulcus in **A)** CT-scan and **B)** T2-weighted MRI scans, respectively.

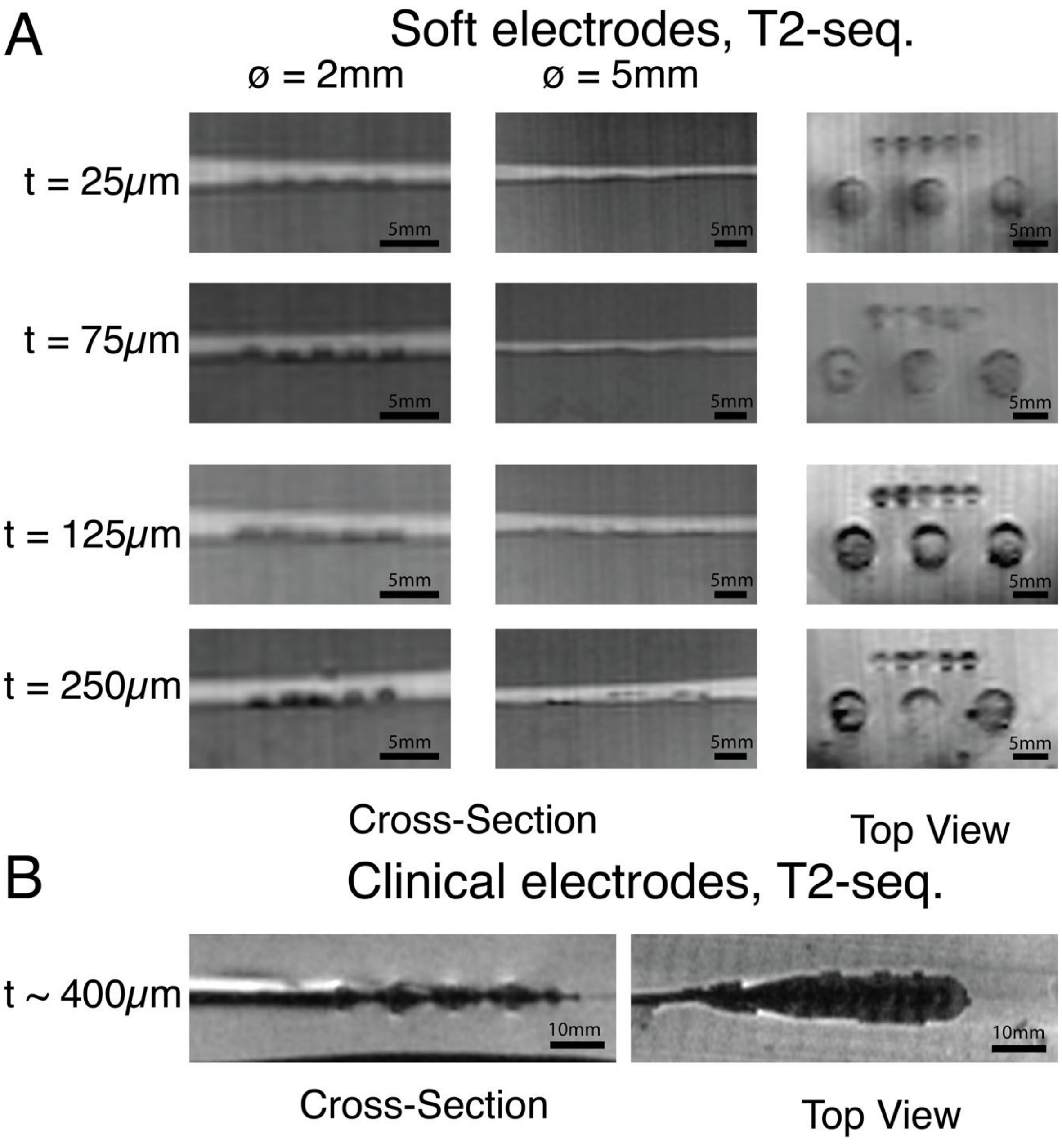

Supplementary Figure *9.* **A)** T2-weighted MRI scan images of soft electrodes in an imaging phantom for different electrode diameters (2mm and 5mm) and thicknesses (25µm, 75µm, 125µm, 250µm). For each case, the cross-section and top view image is presented. B) T2-weighted MRI scan images of clinical electrodes from an ECoG strip (AdTech) in an imaging phantom. The cross-section and top view images are presented.

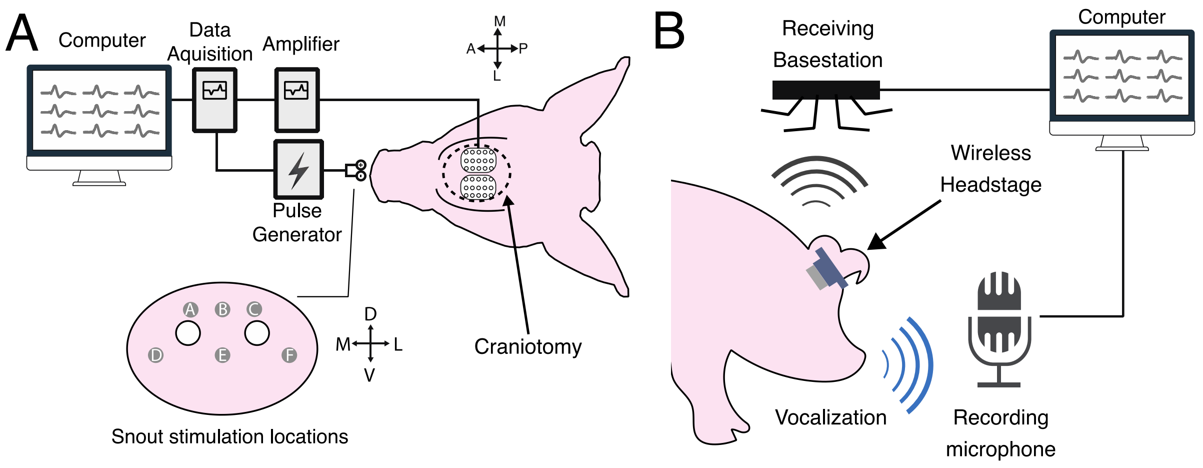

Supplementary Figure 10. Overview of the in vivo experimental setup. **A)** Experimental setup for the somatosensory evoked potentials recordings in the porcine model. An amplifier is connected to the soft electrode arrays in contact with the brain and a pulse stimulator stimulates the groin at different locations. A data acquisition system synchronizes the stimulation with the recording and a central computer station saves and displays the stimulation-locked recordings. **B)** Experimental setup for the subchronic vocalization evoked potentials recordings in the minipig model. A microphone records the vocalization of the freely moving animal. A wireless amplifier headstage transmits the digitized brain recordings to an antenna base station. A central computer station saves and displays the vocalization-locked recordings.

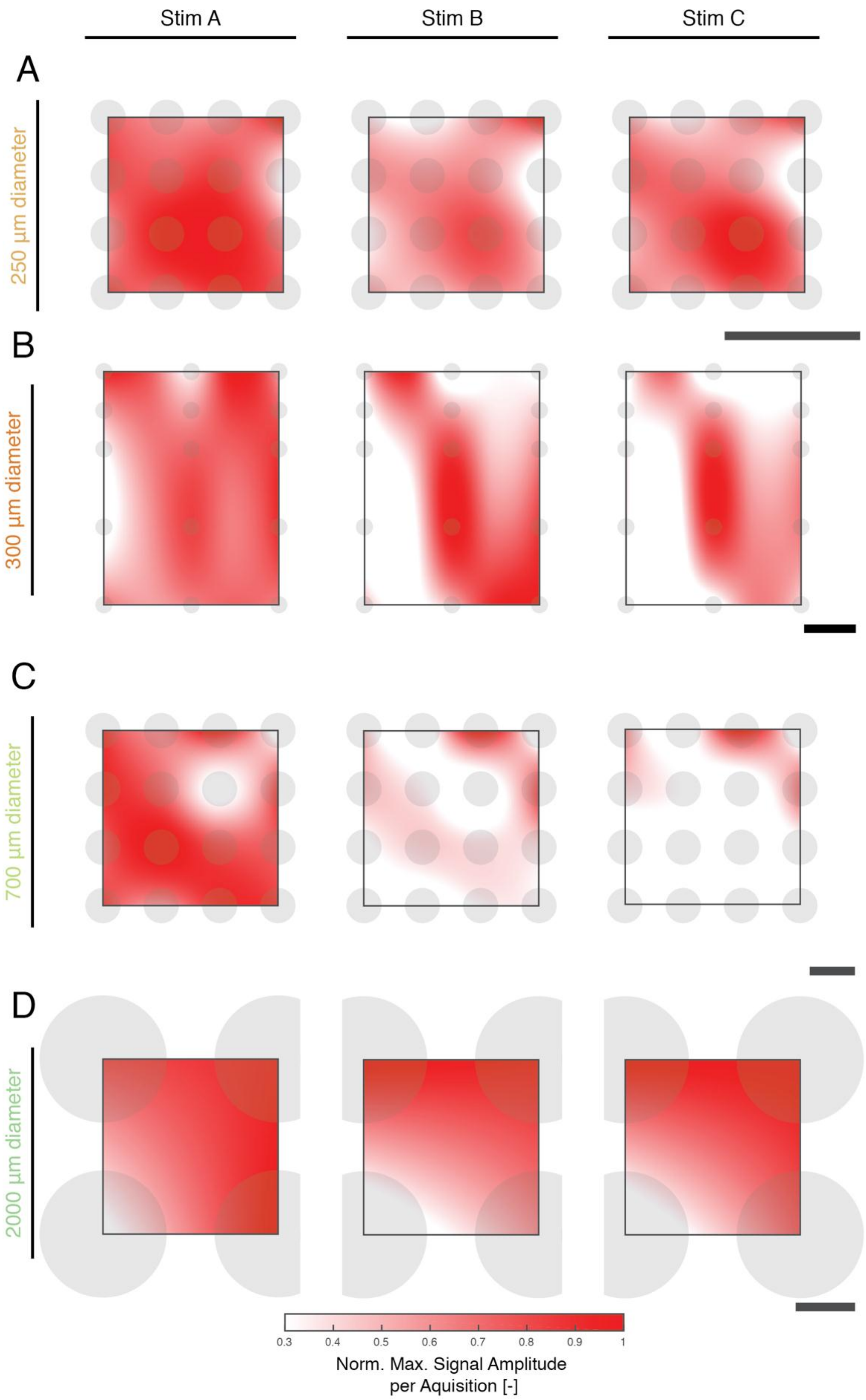

Supplementary Figure 11. A), B), C), D) Activation maps of the recorded somatosensory evoked potentials from different snout stimulation locations and different electrode diameters.

Each grey dot represents the location of the electrode. The color gradient is made from the quadratic interpolation between the electrode locations. The scale bar for each plot is 1mm.

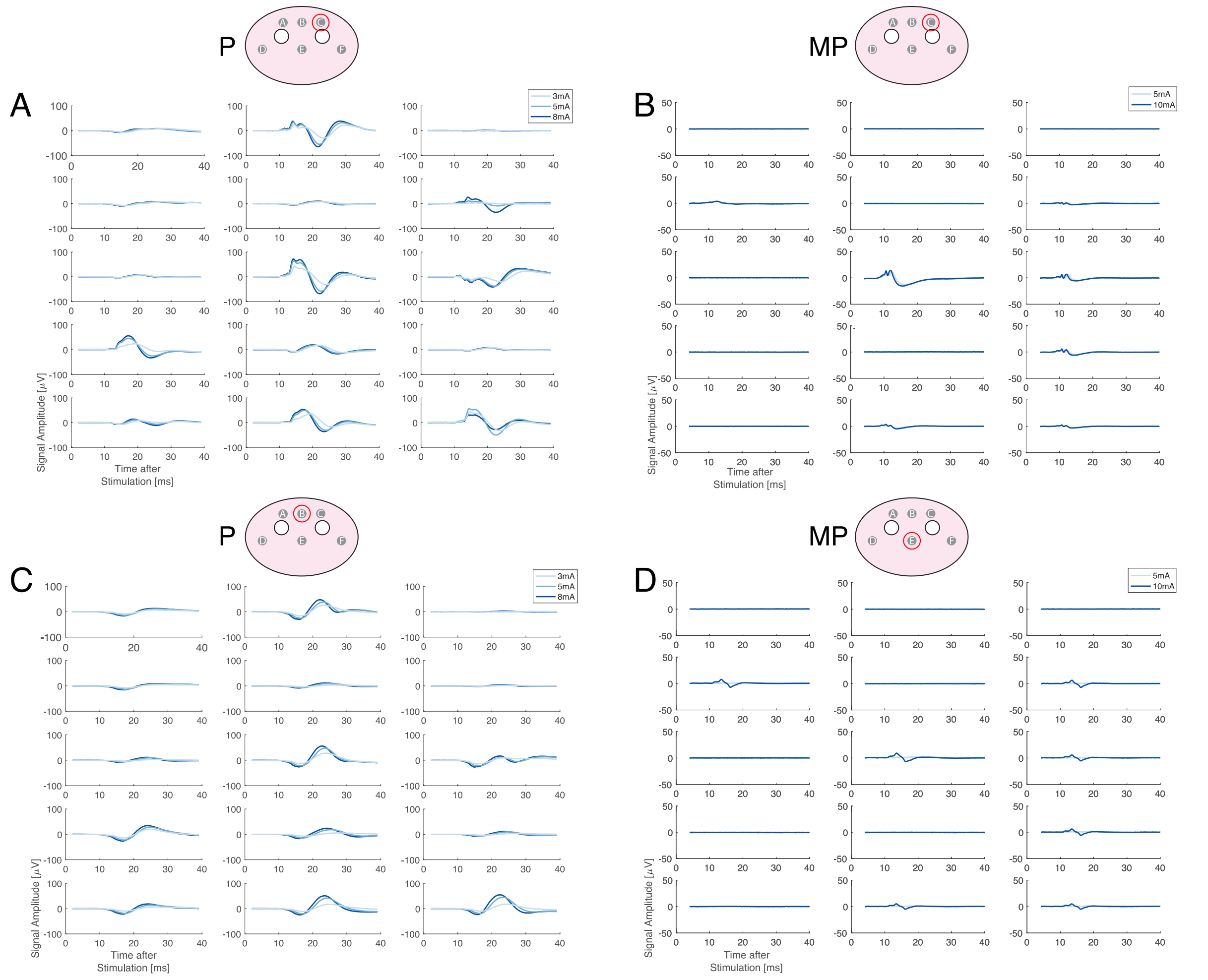

Supplementary Figure 12. Somatosensory evoked potentials recorded by the µECoG (electrode diameter of 300µm) following snout stimulation in a pig (P) and minipig (MP). **A)** Snout stimulation site C in pig. **B)** Snout stimulation site C in minipig. **C)** Snout stimulation site B in pig. **D)** Snout stimulation site E in minipig.

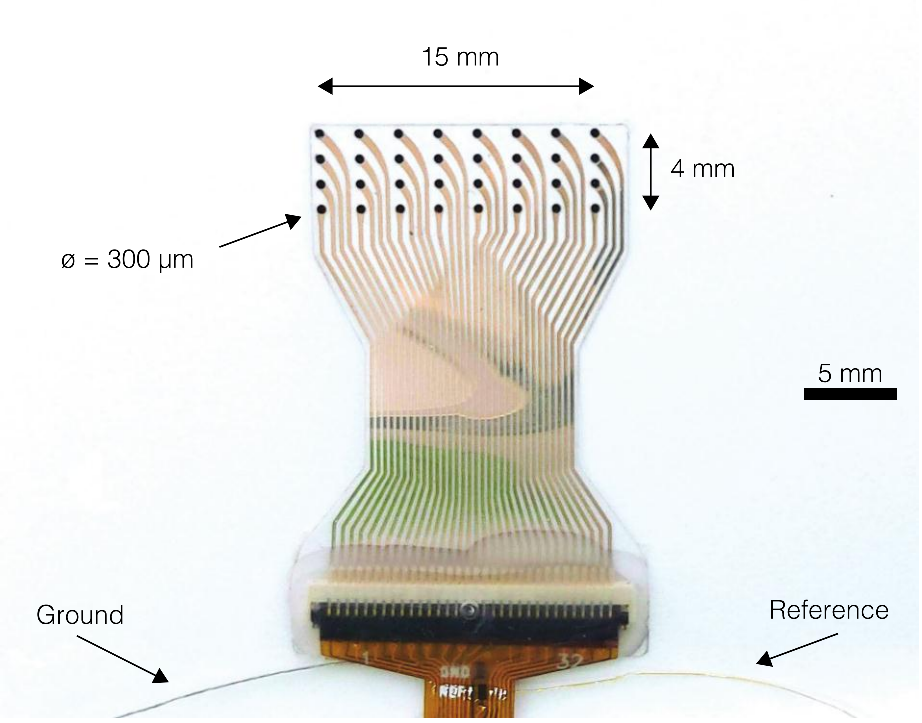

Supplementary Figure 13 32-contact (4x8 grid) soft grid with an electrode diameter of 300µm used for subchronic recording in minipig model.

SIEMENS MAGNETOM Prisma

TA: 4:44 PM: FIX Voxel size: 1.0×1.0×1.0 mmPAT: 2 Rel. SNR: 1.00 : tfl

Properties

Prio recon	Off
Load images to viewer	On
Inline movie	Off
Auto store images	On
Load images to stamp segments	Off
Load images to graphic segments	Off
Auto open inline display	Off
Auto close inline display	Off
Start measurement without further preparation	Off
Wait for user to start	On
Start measurements	Single measurement

Routine

Slab group	1
Slabs	1
Dist. factor	50 %
Position	R1.8 A13.5 F30.5 mm
Orientation	Sagittal
Phase enc. dir.	A >> P
AutoAlign	Head > Brain
Phase oversampling	0 %
Slice oversampling	15.4 %
Slices per slab	208
FoV read	256 mm
FoV phase	100.0 %
Slice thickness	1.00 mm
TR	2300.0 ms
TE	2.25 ms
Averages	1
Concatenations	1
Filter	Prescan Normalize
Coil elements	HC1-7;NC1,2

Contrast - Common

TR	2300.0 ms
TE	2.25 ms
Magn. preparation	Non-sel. IR
TI	900 ms
Flip angle	8 deg
Fat suppr.	None
Water suppr.	None

Contrast - Dynamic

Averages	1
Averaging mode	Long term
Reconstruction	Magnitude
Measurements	1
Multiple series	Each measurement

Resolution - Common

FoV read	256 mm
FoV phase	100.0 %
Slice thickness	1.00 mm
Base resolution	256
Phase resolution	100 %
Slice resolution	100 %
Phase partial Fourier	7/8
Slice partial Fourier	7/8
Interpolation	Off

Resolution - iPAT

PAT mode	GRAPPA
Accel. factor PE	2
Ref. lines PE	24
Accel. factor 3D	1
Reference scan mode	Integrated

Resolution - Filter Image

Image Filter	Off
Distortion Corr.	Off
Prescan Normalize	On
Unfiltered images	Off
Normalize	Off
B1 filter	Off

Resolution - Filter Rawdata

Raw filter	Off
Elliptical filter	Off

Geometry - Common

Slab group	1
Slabs	1
Dist. factor	50 %
Position	R1.8 A13.5 F30.5 mm
Orientation	Sagittal
Phase enc. dir.	A >> P
Slice oversampling	15.4 %
Slices per slab	208
FoV read	256 mm
FoV phase	100.0 %
Slice thickness	1.00 mm
TR	2300.0 ms
Multi-slice mode	Single shot
Series	Ascending
Concatenations	1

Geometry - AutoAlign

Slab group	1
Position	R1.8 A13.5 F30.5 mm
Orientation	Sagittal
Phase enc. dir.	A >> P
AutoAlign	Head > Brain
Initial Position	R1.8 A13.5 F30.5
R	1.8 mm
A	13.5 mm
F	30.5 mm
Initial Rotation	-9.53 deg
Initial Orientation	Sagittal

Geometry - Navigator

System - Miscellaneous

Positioning mode	FIX
Table position	H
Table position	0 mm
MSMA	S - C - T
Sagittal	R >> L
Coronal	A >> P
Transversal	F >> H
Coil Combine Mode	Adaptive Combine
Save uncombined	Off

SIEMENS MAGNETOM Prisma

System - Miscellaneous

Matrix Optimization	Off
AutoAlign	Head > Brain
Coil Select Mode	Off - AutoCoilSelect

System - Adjustments

B0 Shim mode	Tune up
B1 Shim mode	TrueForm
Adjust with body coil	Off
Confirm freq. adjustment	Off
Assume Dominant Fat	Off
Assume Silicone	Off
Adjustment Tolerance	Auto

System - Adjust Volume

Position	Isocenter
Orientation	Transversal
Rotation	0.00 deg
A >> P	263 mm
R >> L	350 mm
F >> H	350 mm
Reset	Off

System - pTx Volumes

B1 Shim mode	TrueForm
Excitation	Non-sel.

System - Tx/Rx

Frequency 1H	123.257291 MHz
Correction factor	1
Gain	Low
Img. Scale Cor.	1.000
Reset	Off
? Ref. amplitude 1H	0.000 V

Physio - Signal1

1st Signal/Mode	None
TR	2300.0 ms
Concatenations	1

Physio - Cardiac

Magn. preparation	Non-sel. IR
TI	900 ms
Fat suppr.	None
Dark blood	Off
FoV read	256 mm
FoV phase	100.0 %
Phase resolution	100 %

Physio - PACE

Resp. control	Off
Concatenations	1

Inline - Common

Subtract	Off
Measurements	1
StdDev	Off
Save original images	On

Inline - MIP

MIP-Sag	Off
MIP-Cor	Off
MIP-Tra	Off
MIP-Time	Off

Inline - MIP

Save original images	On

Inline - Composing

Distortion Corr.	Off

Sequence - Part 1

Introduction	On
Dimension	3D
Elliptical scanning	Off
Reordering	Linear
Asymmetric echo	Allowed
Flow comp.	No
Multi-slice mode	Single shot
Echo spacing	6.8 ms
Bandwidth	200 Hz/Px

Sequence - Part 2

RF pulse type	Normal
Gradient mode	Normal
Excitation	Non-sel.
RF spoiling	On
Incr. Gradient spoiling	Off
Turbo factor	210

Sequence - Assistant

Mode	Off

Supplementary Figure 14. *MRI sequence parameters for the T1-weighted sequence*

SIEMENS MAGNETOM Prisma

TA: 6:56 PM: FIX Voxel size: 0.5×0.5×0.5 mmPAT: Off Rel. SNR: 1.00 : spcR

Properties

Prio recon	Off
Load images to viewer	On
Inline movie	Off
Auto store images	On
Load images to stamp segments	Off
Load images to graphic segments	Off
Auto open inline display	Off
Auto close inline display	Off
Start measurement without further preparation	Off
Wait for user to start	Off
Start measurements	Single measurement

Routine

Slab group	1
Slabs	1
Position	R12.7 A28.1 F0.9 mm
Orientation	S > C-29.2 > T-16.8
Phase enc. dir.	A >> P
AutoAlign	---
Phase oversampling	10 %
Slice oversampling	16.7 %
Slices per slab	96
FoV read	153 mm
FoV phase	100.0 %
Slice thickness	0.50 mm
TR	1400 ms
TE	98 ms
Averages	1.7
Concatenations	1
Filter	Raw filter, Prescan Normalize
Coil elements	HC1-7;NC1,2

Contrast - Common

TR	1400 ms
TE	98 ms
MTC	Off
Magn. preparation	None
Flip angle	120 deg
Fat suppr.	None
Blood suppr.	Off
Restore magn.	On

Contrast - Dynamic

Averages	1.7
Reconstruction	Magnitude
Measurements	1
Multiple series	Each measurement

Resolution - Common

FoV read	153 mm
FoV phase	100.0 %
Slice thickness	0.50 mm
Base resolution	320
Phase resolution	100 %
Slice resolution	50 %
Phase partial Fourier	Allowed
Slice partial Fourier	7/8
Interpolation	Off

Resolution - iPAT

PAT mode	None

Resolution - Filter Image

Image Filter	Off
Distortion Corr.	Off
Prescan Normalize	On
Unfiltered images	Off
Normalize	Off
B1 filter	Off

Resolution - Filter Rawdata

Raw filter	On
Elliptical filter	Off

Geometry - Common

Slab group	1
Slabs	1
Position	R12.7 A28.1 F0.9 mm
Orientation	S > C-29.2 > T-16.8
Phase enc. dir.	A >> P
Slice oversampling	16.7 %
Slices per slab	96
FoV read	153 mm
FoV phase	100.0 %
Slice thickness	0.50 mm
TR	1400 ms
Series	Ascending
Concatenations	1

Geometry - AutoAlign

Slab group	1
Position	R12.7 A28.1 F0.9 mm
Orientation	S > C-29.2 > T-16.8
Phase enc. dir.	A >> P
AutoAlign	---
Initial Position	R12.7 A28.1 F0.9
R	12.7 mm
A	28.1 mm
F	0.9 mm
Initial Rotation	0.00 deg
Initial Orientation	S > C
S > C	-29.2
> T	-16.8

Geometry - Saturation

Fat suppr.	None
Restore magn.	On
Special sat.	None

Geometry - Navigator

System - Miscellaneous

Positioning mode	FIX
Table position	H
Table position	0 mm
MSMA	S - C - T
Sagittal	R >> L
Coronal	A >> P
Transversal	F >> H
Coil Combine Mode	Adaptive Combine

SIEMENS MAGNETOM Prisma

System - Miscellaneous

Save uncombined	Off
Matrix Optimization	Performance
AutoAlign	---
Coil Select Mode	Off - AutoCoilSelect

System - Adjustments

B0 Shim mode	Tune up
B1 Shim mode	Volume-selective
Adjust with body coil	Off
Confirm freq. adjustment	Off
Assume Dominant Fat	Off
Assume Silicone	Off
Adjustment Tolerance	Auto

System - Adjust Volume

Position	Isocenter
Orientation	Transversal
Rotation	0.00 deg
A >> P	263 mm
R >> L	350 mm
F >> H	350 mm
Reset	Off

System - pTx Volumes

B1 Shim mode	Volume-selective
Excitation	Slab-sel.
pTx Volume	1
Vol. Property	B1 Shim Vol.
Position	R24.5 A4.0 F3.5 mm
Orientation	C > S33.5 > T-2.6
Rotation	-70.69 deg
F >> H	68 mm
R >> L	55 mm
A >> P	51 mm
Vol. Visibility	On

System - Tx/Rx

Frequency 1H	123.257291 MHz
Correction factor	1
Gain	High
Img. Scale Cor.	1.000
Reset	Off
? Ref. amplitude 1H	0.000 V

Physio - Signal1

1st Signal/Mode	None
Trigger delay	0 ms
TR	1400 ms
Concatenations	1

Physio - Cardiac

Magn. preparation	None
Fat suppr.	None
Dark blood	Off
FoV read	153 mm
FoV phase	100.0 %
Phase resolution	100 %

Physio - PACE

Resp. control	Off
Concatenations	1

Inline - Common

Subtract	Off
Measurements	1
StdDev	Off
Save original images	On

Inline - MIP

MIP-Sag	Off
MIP-Cor	Off
MIP-Tra	Off
MIP-Time	Off
Save original images	On

Inline - Composing

Distortion Corr.	Off

Sequence - Part 1

Introduction	On
Dimension	3D
Elliptical scanning	Off
Reordering	Linear
Flow comp.	No
Echo spacing	6.7 ms
Adiabatic-mode	Off
Bandwidth	289 Hz/Px

Sequence - Part 2

Echo train duration	422 ms
RF pulse type	Normal
Gradient mode	Fast
Excitation	Slab-sel.
Flip angle mode	Constant
Turbo factor	98

Sequence - Assistant

Allowed delay	30 s

Supplementary Figure 15. MRI sequence parameters for the T2-weighted sequence

SIEMENS MAGNETOM TrioTim syngo MR B17

\\USER\fMRI_ARCHIVE\HOLLOW\HOLLOW\T1

TA: 5:04 PAT: 2 Voxel size: 1.0×1.0×1.0 mm Rel. SNR: 1.00 SIEMENS: tfl

Properties

Prio Recon	Off
Before measurement	
After measurement	
Load to viewer	On
Inline movie	Off
Auto store images	On
Load to stamp segments	On
Load images to graphic segments	Off
Auto open inline display	Off
Start measurement without further preparation	On
Wait for user to start	On
Start measurements	single

Routine

Slab group 1	
Slabs	1
Dist. factor	50 %
Position	L0.0 A27.0 F16.2
Orientation	Sagittal
Phase enc. dir.	A >> P
Rotation	0.00 deg
Phase oversampling	15 %
Slice oversampling	16.7 %
Slices per slab	192
FoV read	256 mm
FoV phase	100.0 %
Slice thickness	1.00 mm
TR	1900 ms
TE	2.27 ms
Averages	1
Concatenations	1
Filter	Prescan Normalize
Coil elements	HEA;HEP

Contrast

Magn. preparation	Non-sel. IR
TI	900 ms
Flip angle	9 deg
Fat suppr.	None
Water suppr.	None
Averaging mode	Long term
Reconstruction	Magnitude
Measurements	1
Multiple series	Each measurement

Resolution

Base resolution	256
Phase resolution	100 %
Slice resolution	100 %
Phase partial Fourier	Off
Slice partial Fourier	Off
Interpolation	Off
PAT mode	GRAPPA
Accel. factor PE	2
Ref. lines PE	24
Accel. factor 3D	1
Matrix Coil Mode	Auto (Triple)
Reference scan mode	Integrated
Image Filter	Off
Distortion Corr.	Off
Unfiltered images	Off
Prescan Normalize	On
Normalize	Off
B1 filter	Off
Raw filter	Off
Elliptical filter	Off

Geometry

Multi-slice mode	Single shot
Series	Ascending

System

Body	Off
HEP	On
HEA	On
SP4	Off
SP2	Off
SP8	Off
SP6	Off
SP3	Off
SP1	Off
SP7	Off
SP5	Off
Positioning mode	FIX
Table position	H
Table position	0 mm
MSMA	S - C - T
Sagittal	R >> L
Coronal	A >> P
Transversal	F >> H
Save uncombined	Off
Coil Combine Mode	Adaptive Combine
AutoAlign	---
Auto Coil Select	Default
Shim mode	Tune up
Adjust with body coil	On
Confirm freq. adjustment	Off
Assume Silicone	Off
? Ref. amplitude 1H	0.000 V
Adjustment Tolerance	Auto
Adjust volume	
Position	Isocenter
Orientation	Transversal
Rotation	0.00 deg
R >> L	350 mm
A >> P	263 mm
F >> H	350 mm

Physio

1st Signal/Mode	None
Dark blood	Off
Resp. control	Off

Inline

Subtract	Off
Std-Dev-Sag	Off
Std-Dev-Cor	Off
Std-Dev-Tra	Off
Std-Dev-Time	Off
MIP-Sag	Off
MIP-Cor	Off
MIP-Tra	Off
MIP-Time	Off

SIEMENS MAGNETOM TrioTim syngo MR B17

Save original images	On
**Sequence**	
Introduction	On
Dimension	3D
Elliptical scanning	Off
Asymmetric echo	Allowed
Bandwidth	190 Hz/Px
Flow comp.	No
Echo spacing	6.7 ms
RF pulse type	Normal
Gradient mode	Fast*
Excitation	Non-sel.
RF spoiling	On

Supplementary Figure 16 MRI sequence parameters for the T1-weighted sequence on Siemens Magnetom 3T

SIEMENS MAGNETOM TrioTim syngo MR B17

\\USER\fMRI_ARCHIVE\HOLLOW\HOLLOW\T2

TA: 5:09 PAT: 2 Voxel size: 1.0×1.0×1.0 mm Rel. SNR: 1.00 SIEMENS: tse_vfl

Properties	
Prio Recon	Off
Before measurement	
After measurement	
Load to viewer	On
Inline movie	Off
Auto store images	On
Load to stamp segments	Off
Load images to graphic segments	Off
Auto open inline display	Off
Start measurement without further preparation	On
Wait for user to start	On
Start measurements	single

Routine	
Slab group 1	
Slabs	1
Position	L0.0 A27.0 F16.2
Orientation	Sagittal
Phase enc. dir.	A >> P
Rotation	0.00 deg
Phase oversampling	0 %
Slice oversampling	0.0 %
Slices per slab	192
FoV read	256 mm
FoV phase	100.0 %
Slice thickness	1.00 mm
TR	3200 ms
TE	408 ms
Averages	1.0
Concatenations	1
Filter	Raw filter, Prescan Normalize
Coil elements	HEA;HEP

Contrast	
MTC	Off
Magn. preparation	None
Fat suppr.	None
Water suppr.	None
Restore magn.	Off
Reconstruction	Magnitude
Measurements	1
Multiple series	Each measurement

Resolution	
Base resolution	256
Phase resolution	100 %
Slice resolution	100 %
Phase partial Fourier	Allowed
Slice partial Fourier	Off
Interpolation	Off
PAT mode	GRAPPA
Accel. factor PE	2
Ref. lines PE	24
Accel. factor 3D	1
Matrix Coil Mode	Auto (Triple)
Reference scan mode	Integrated
Image Filter	Off
Distortion Corr.	Off
Unfiltered images	Off
Prescan Normalize	On
Normalize	Off
B1 filter	Off
Raw filter	On
Intensity	Weak
Slope	25
Elliptical filter	Off

Geometry	
Special sat.	None

System	
Body	Off
HEP	On
HEA	On
SP4	Off
SP2	Off
SP8	Off
SP6	Off
SP3	Off
SP1	Off
SP7	Off
SP5	Off
Positioning mode	FIX
Table position	H
Table position	0 mm
MSMA	S - C - T
Sagittal	R >> L
Coronal	A >> P
Transversal	F >> H
Save uncombined	Off
Coil Combine Mode	Adaptive Combine
AutoAlign	---
Auto Coil Select	Default
Shim mode	Standard
Adjust with body coil	Off
Confirm freq. adjustment	Off
Assume Silicone	Off
? Ref. amplitude 1H	0.000 V
Adjustment Tolerance	Auto
Adjust volume	
Position	L0.0 A27.0 F16.2
Orientation	Sagittal
Rotation	0.00 deg
F >> H	256 mm
A >> P	256 mm
R >> L	192 mm

Physio	
1st Signal/Mode	None
Dark blood	Off
Resp. control	Off

Inline	
Subtract	Off
Std-Dev-Sag	Off
Std-Dev-Cor	Off
Std-Dev-Tra	Off
Std-Dev-Time	Off
MIP-Sag	Off
MIP-Cor	Off
MIP-Tra	Off
MIP-Time	Off
Save original images	On

3/+

SIEMENS MAGNETOM TrioTim syngo MR B17

Sequence	
Introduction	On
Dimension	3D
Bandwidth	751 Hz/Px
Flow comp.	No
Allowed delay	30 s
Echo spacing	3.36 ms
Adiabatic-mode	Off
Define	Echo trains
Turbo factor	141
Slice turbo factor	2
Echo trains per slice	1
Echo train duration	877
RF pulse type	Normal
Gradient mode	Fast
Excitation	Non-sel.
Flip angle mode	T2 var

4/+

Supplementary Figure 17 MRI sequence parameters for the T2-weighted sequence on Siemens Magnetom 3T

SIEMENS MAGNETOM Prisma

\\EPFL\S.Lacour\Elvira\SL_ECoGarrays\t1_tse_sag_p2_180mm_2mm *
TA: 2:55 PM: REF Voxel size: 0.4×0.4×2.0 mmPAT: 2 Rel. SNR: 1.00 : tse

Properties

Prio recon	Off
Load images to viewer	On
Inline movie	Off
Auto store images	On
Load images to stamp segments	Off
Load images to graphic segments	Off
Auto open inline display	Off
Auto close inline display	Off
Start measurement without further preparation	Off
Wait for user to start	On
Start measurements	Single measurement

Routine

Slice group	1
Slices	24
Dist. factor	20 %
Position	R1.7 P1.4 F14.6 mm
Orientation	Sagittal
Phase enc. dir.	A >> P
AutoAlign	---
Phase oversampling	40 %
FoV read	180 mm
FoV phase	100.0 %
Slice thickness	2.0 mm
TR	1190.0 ms
TE	6.7 ms
Averages	3
Concatenations	2
Filter	Prescan Normalize, Elliptical filter
Coil elements	HC1-7;NC1,2

Contrast - Common

TR	1190.0 ms
TE	6.7 ms
TD	0.0 ms
MTC	Off
Magn. preparation	None
Flip angle	150 deg
Fat suppr.	None
Water suppr.	None
Restore magn.	Off

Contrast - Dynamic

Averages	3
Averaging mode	Long term
Reconstruction	Magnitude
Measurements	1
Multiple series	Each measurement

Resolution - Common

FoV read	180 mm
FoV phase	100.0 %
Slice thickness	2.0 mm
Base resolution	256
Phase resolution	80 %
Phase partial Fourier	Off
Trajectory	Cartesian
Interpolation	On

Resolution - iPAT

PAT mode	GRAPPA
Accel. factor PE	2
Ref. lines PE	32
Reference scan mode	Self-calibration

Resolution - Filter Image

Image Filter	Off
Distortion Corr.	Off
Prescan Normalize	On
Unfiltered images	Off
Normalize	Off
B1 filter	Off

Resolution - Filter Rawdata

Raw filter	Off
Elliptical filter	On

Geometry - Common

Slice group	1
Slices	24
Dist. factor	20 %
Position	R1.7 P1.4 F14.6 mm
Orientation	Sagittal
Phase enc. dir.	A >> P
FoV read	180 mm
FoV phase	100.0 %
Slice thickness	2.0 mm
TR	1190.0 ms
Multi-slice mode	Interleaved
Series	Interleaved
Concatenations	2

Geometry - AutoAlign

Slice group	1
Position	R1.7 P1.4 F14.6 mm
Orientation	Sagittal
Phase enc. dir.	A >> P
AutoAlign	---
Initial Position	R1.7 P1.4 F14.6
R	1.7 mm
P	1.4 mm
F	14.6 mm
Initial Rotation	0.00 deg
Initial Orientation	Sagittal

Geometry - Saturation

Sat. region	1
Thickness	80 mm
Position	L1.1 A111.9 F15.4 mm
Orientation	C > T-7.8 > S0.5
Shape	Standard
Water suppr.	None
Restore magn.	Off
Special sat.	None

Geometry - Navigator

System - Miscellaneous

Positioning mode	REF
Table position	H

System - Miscellaneous

Table position	0 mm
MSMA	S - C - T
Sagittal	R >> L
Coronal	A >> P
Transversal	F >> H
Coil Combine Mode	Adaptive Combine
Save uncombined	Off
Matrix Optimization	Off
AutoAlign	---
Coil Select Mode	Off - AutoCoilSelect

System - Adjustments

B0 Shim mode	Tune up
B1 Shim mode	TrueForm
Adjust with body coil	Off
Confirm freq. adjustment	Off
Assume Dominant Fat	Off
Assume Silicone	Off
Adjustment Tolerance	Auto

System - Adjust Volume

Position	Isocenter
Orientation	Transversal
Rotation	0.00 deg
A >> P	263 mm
R >> L	350 mm
F >> H	350 mm
Reset	Off

System - pTx Volumes

B1 Shim mode	TrueForm

System - Tx/Rx

Frequency 1H	123.257291 MHz
Correction factor	1
Gain	High
Img. Scale Cor.	1.000
Reset	Off
? Ref. amplitude 1H	0.000 V

Physio - Signal1

1st Signal/Mode	None
TR	1190.0 ms
Concatenations	2

Physio - Cardiac

Magn. preparation	None
Fat suppr.	None
Dark blood	Off
FoV read	180 mm
FoV phase	100.0 %
Phase resolution	80 %
Trajectory	Cartesian

Physio - PACE

Resp. control	Off
Concatenations	2

Inline - Common

Subtract	Off
Measurements	1
StdDev	Off
Save original images	On

Inline - MIP

MIP-Sag	Off
MIP-Cor	Off
MIP-Tra	Off
MIP-Time	Off
Save original images	On

Inline - Composing

Distortion Corr.	Off

Sequence - Part 1

Introduction	On
Dimension	2D
Compensate T2 decay	Off
Reduce Motion Sens.	On
Contrasts	1
Flow comp.	No
Multi-slice mode	Interleaved
Free echo spacing	Off
Echo spacing	6.65 ms
Bandwidth	399 Hz/Px

Sequence - Part 2

Define	Turbo factor
Echo trains per slice	24
Phase correction	Automatic
Acoustic noise reduction	None
RF pulse type	Fast
Gradient mode	Fast
Hyperecho	Off
WARP	Off
Red. EC sensitivity	Off
Turbo factor	6

Sequence - Assistant

Mode	Min flip angle
Min flip angle	130 deg
Allowed delay	60 s

Supplementary Figure 18 MRI sequence parameters for the T2 turbo spin echo sequence

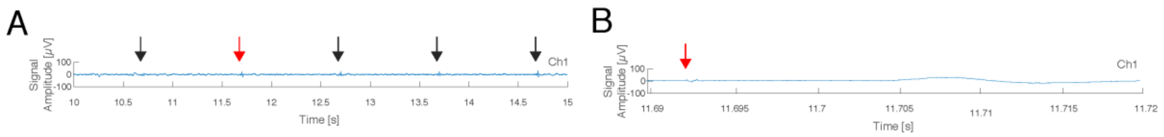

Supplementary Figure 19 Example of raw recording for 5 mm diameter electrode, with stimulation site C at 5 mA current amplitude. **A)** Recording over multiple stimulation pulses, indicated by the arrows at the top. **B)** Highlight of a single recording, with the arrow signaling the timing of the snout stimulation. The red arrow represents the position in time relative to A)

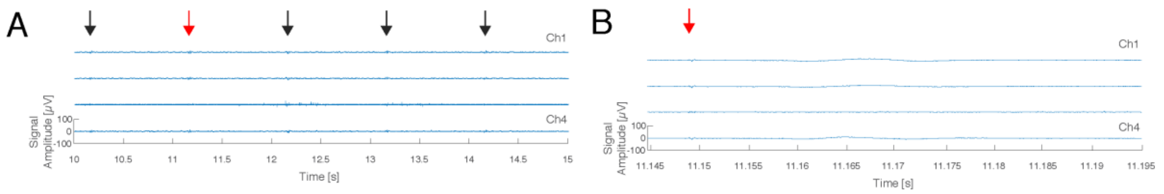

Supplementary Figure 20 Example of raw recording for 2 mm diameter electrode, with stimulation site C at 5 mA current amplitude. **A)** Recording over multiple stimulation pulses, indicated by the arrows at the top. **B)** Highlight of a single recording, with the arrow signaling the timing of the snout stimulation. The red arrow represents the position in time relative to A)

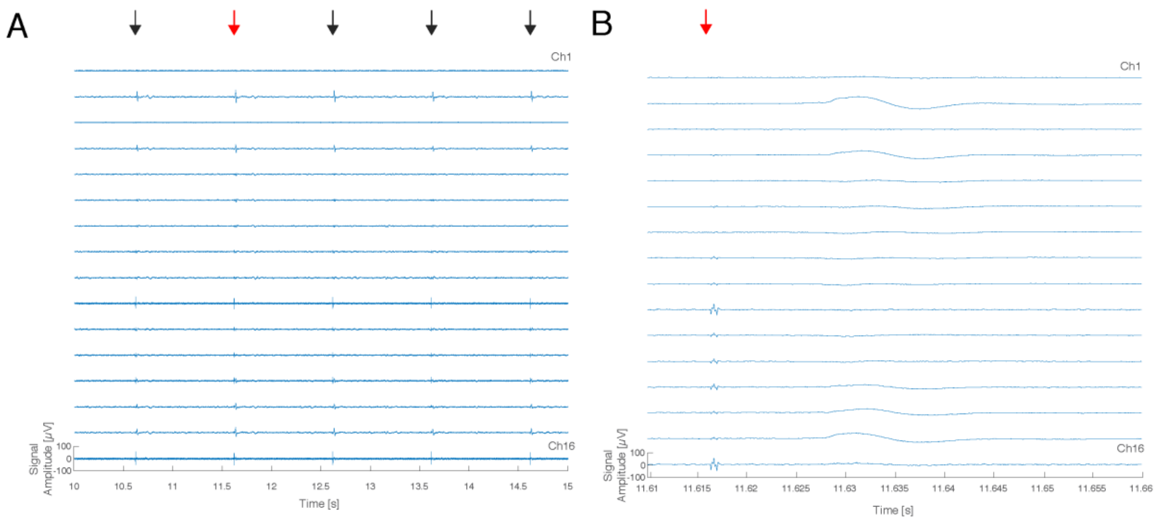

Supplementary Figure 21 Example of raw recording for 700 µm diameter electrode, with stimulation site C at 5 mA current amplitude. **A)** Recording over multiple stimulation pulses, indicated by the arrows at the top. **B)** Highlight of a single recording, with the arrow signaling

the timing of the snout stimulation. The red arrow represents the position in time relative to A)

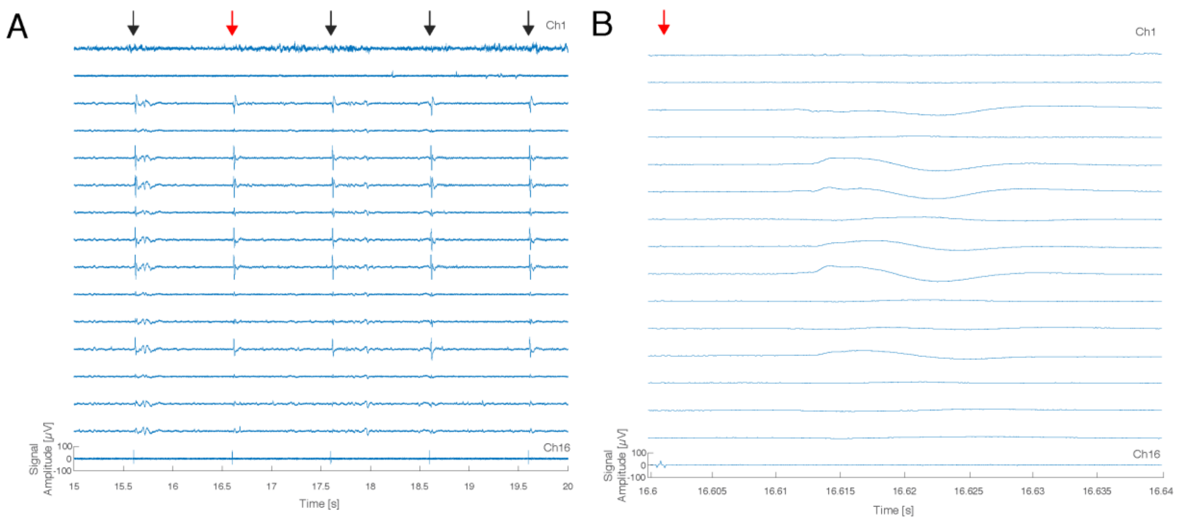

Supplementary Figure 22 Example of raw recording for 300 µm diameter electrode, with stimulation site C at 5 mA current amplitude. **A)** Recording over multiple stimulation pulses, indicated by the arrows at the top. **B)** Highlight of a single recording, with the arrow signaling the timing of the snout stimulation. The red arrow represents the position in time relative to A)

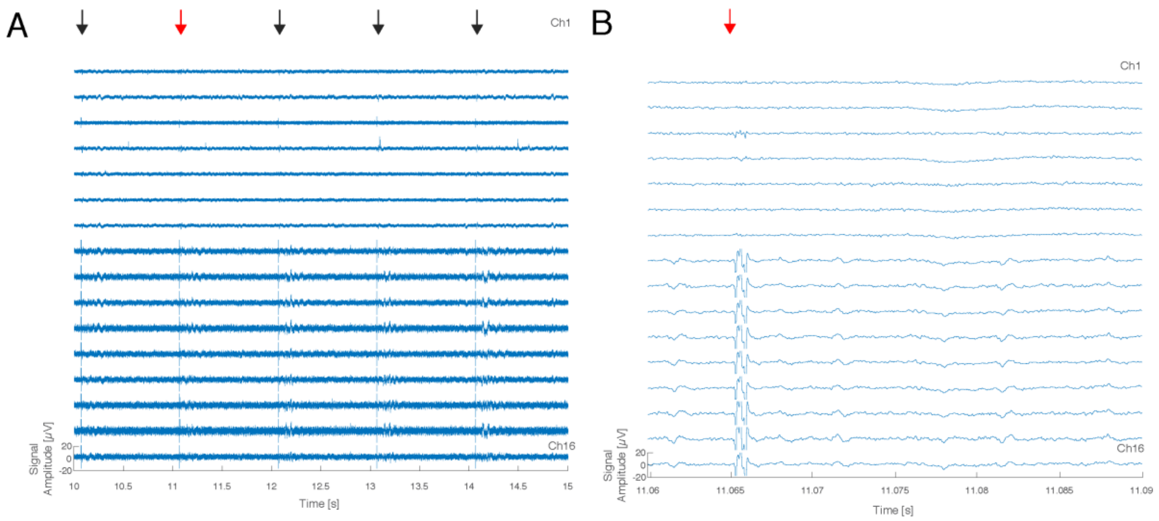

Supplementary Figure 23 Example of raw recording for 250 µm diameter electrode, with stimulation site C at 5 mA current amplitude. **A)** Recording over multiple stimulation pulses, indicated by the arrows at the top. **B)** Highlight of a single recording, with the arrow signaling

the timing of the snout stimulation. The red arrow represents the position in time relative to A)

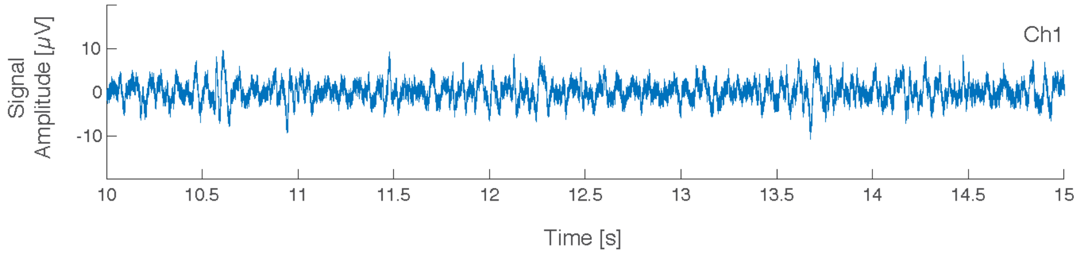

Supplementary Figure 24 Baseline recording with 5 mm electrode diameter.

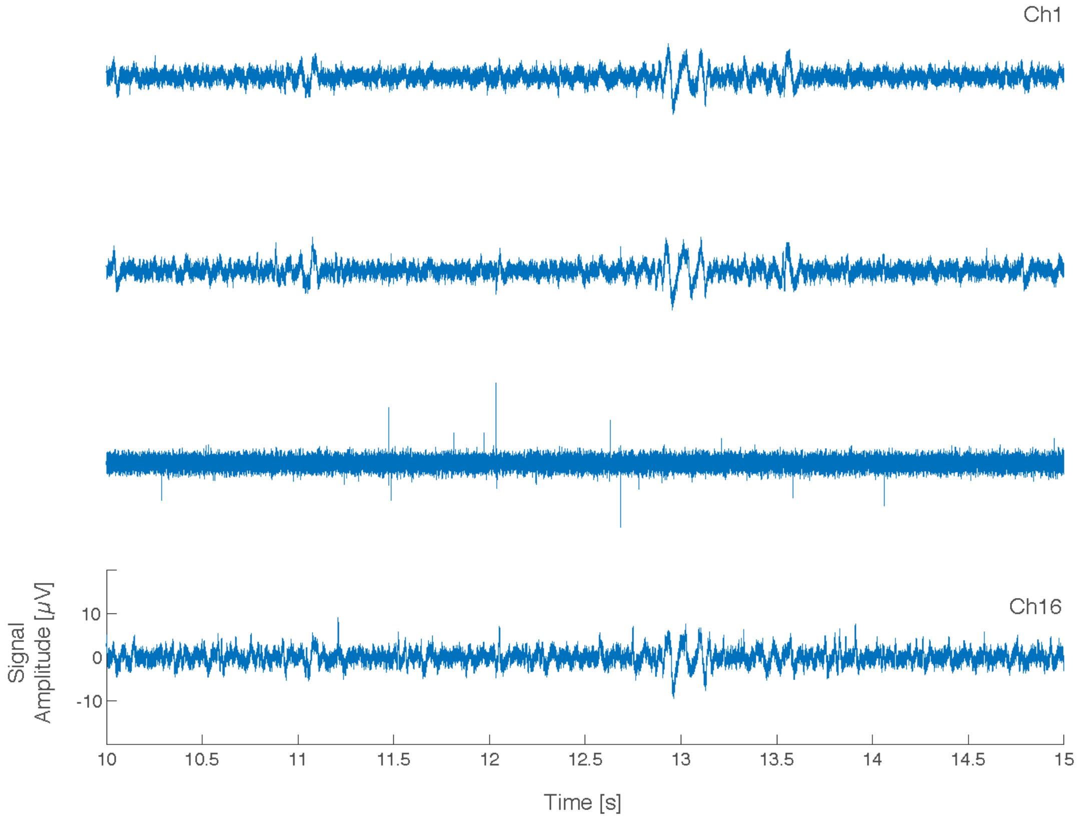

Supplementary Figure 25 Baseline recording with 2 mm electrode diameter.

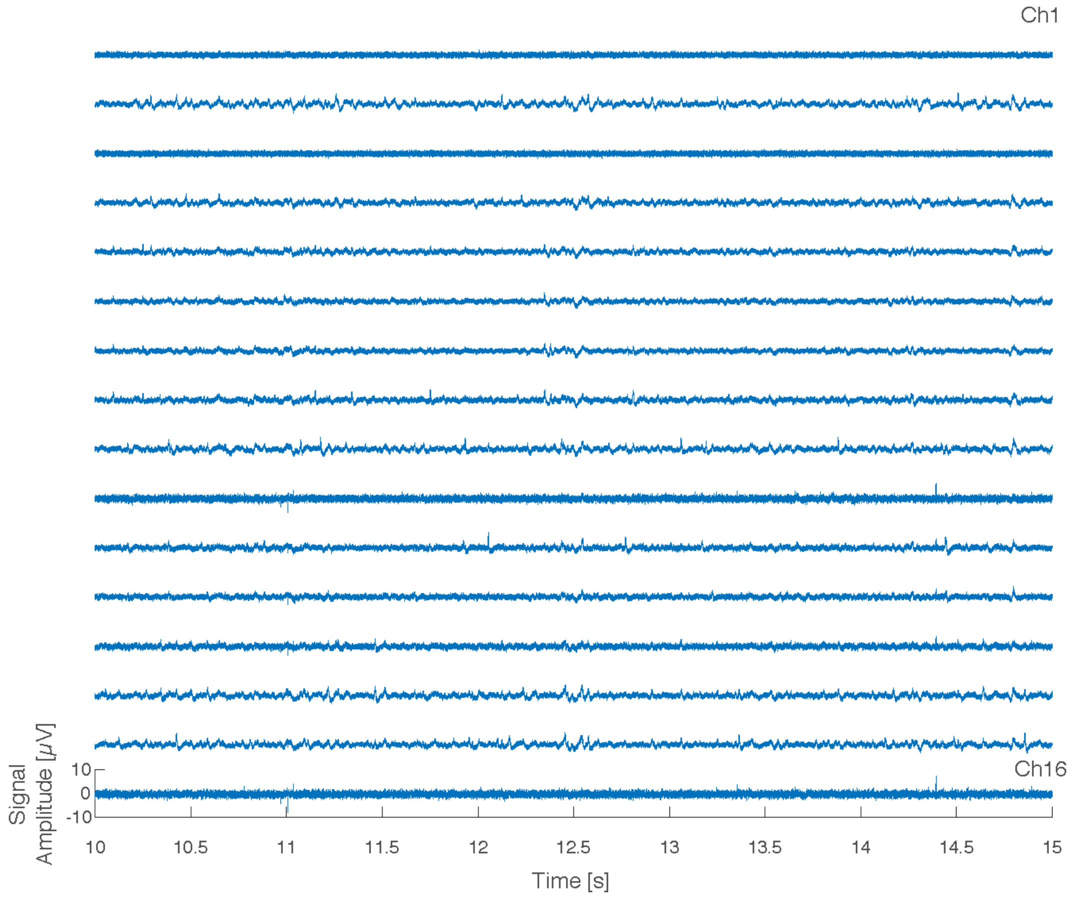

Supplementary Figure 26 Baseline recording with 700 µm electrode diameter.

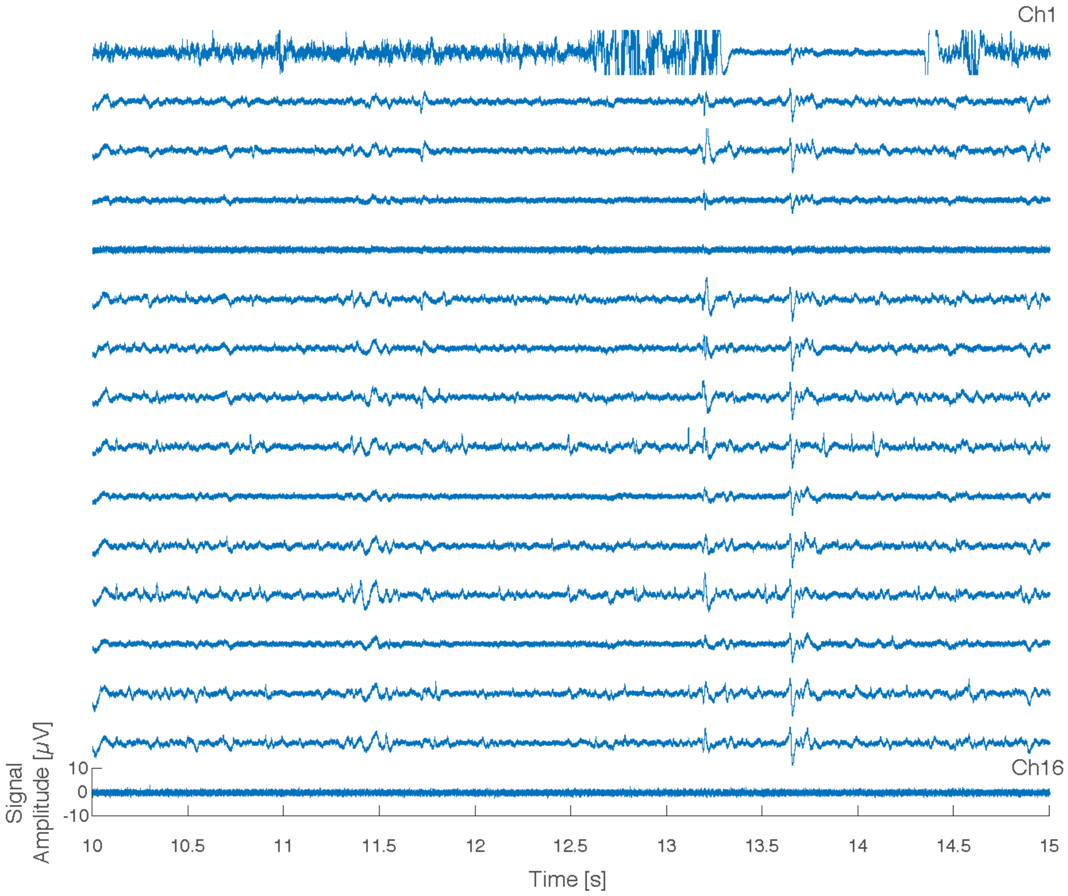

Supplementary Figure 27 Baseline recording with 300 µm electrode diameter.

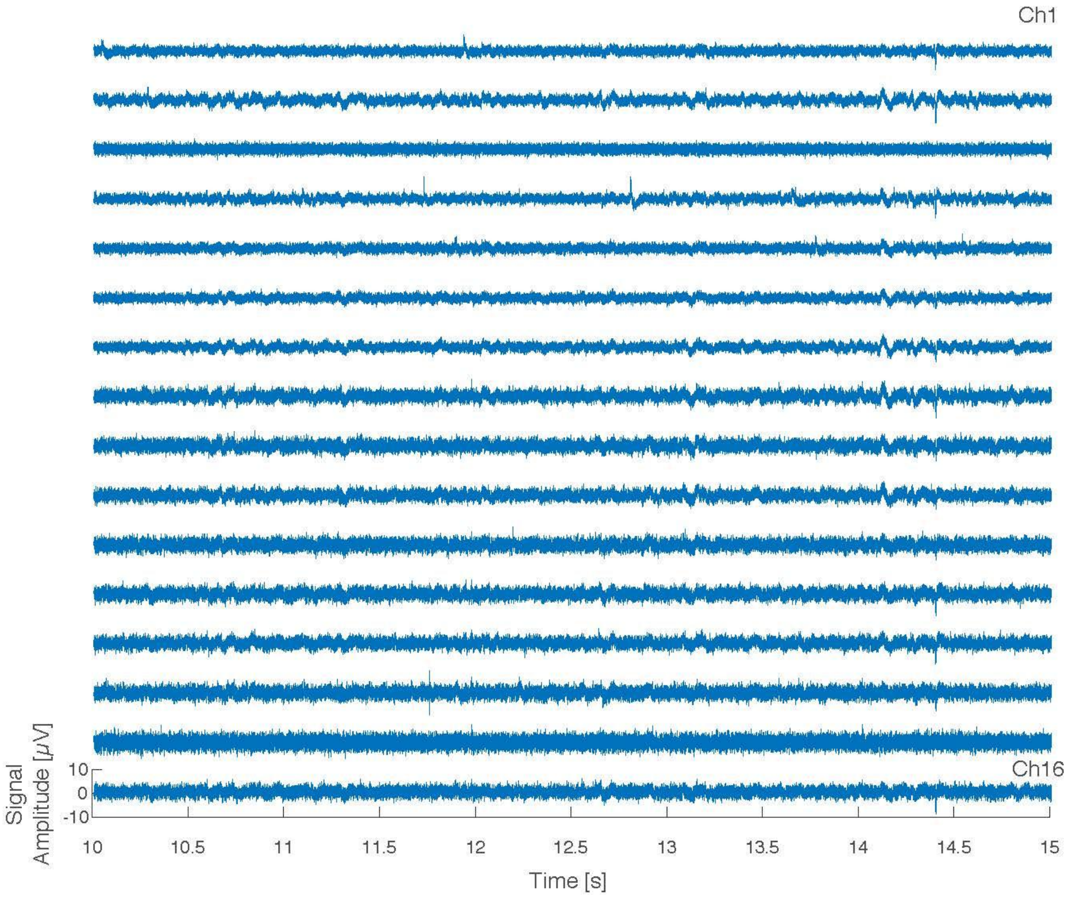

Supplementary Figure 28 Baseline recording with 250 µm electrode diameter.

Bibliography

[1] D. A. Moses, M. K. Leonard, J. G. Makin, E. F. Chang, *Nat. Commun.* **2019**, *10*, DOI 10.1038/s41467-019-10994-4.

[2] D. Khodagholy, J. N. Gelinas, T. Thesen, W. Doyle, O. Devinsky, G. G. Malliaras, G. Buzsáki, *Nat. Neurosci.* **2015**, *18*, 310.

[3] D. Khodagholy, J. N. Gelinas, Z. Zhao, M. Yeh, M. Long, J. D. Greenlee, W. Doyle, O. Devinsky, G. Buzsáki, *Sci. Adv.* **2016**, *2*, 1.

[4] I. D. Johnston, D. K. McCluskey, C. K. L. Tan, M. C. Tracey, *J. Micromechanics Microengineering* **2014**, *24*, DOI 10.1088/0960-1317/24/3/035017.

[5] Dupont, “Dupont ™ kapton ® summary of properties,” **2017**.
